# ZLN005 improves the survival of polymicrobial sepsis by increasing the bacterial killing *via* inducing lysosomal acidification and biogenesis in phagocytes

**DOI:** 10.3389/fimmu.2023.1089905

**Published:** 2023-02-03

**Authors:** Yosuke Suzuki, Daisuke Kami, Toshihiko Taya, Arata Sano, Takehiro Ogata, Satoaki Matoba, Satoshi Gojo

**Affiliations:** ^1^ Department of Cardiovascular Medicine, Graduate School of Medicine, Kyoto Prefectural University of Medicine, Kyoto, Japan; ^2^ Department of Regenerative Medicine, Graduate School of Medicine, Kyoto Prefectural University of Medicine, Kyoto, Japan; ^3^ Department of Pathology and Cell Regulation, Graduate School of Medicine, Kyoto Prefectural University of Medicine, Kyoto, Japan

**Keywords:** lysosome acidity, lysosomal biogenesis, bacterial degradation, mitochondrial biogenesis, sepsis

## Abstract

Polymicrobial sepsis still has a high mortality rate despite the development of antimicrobial agents, elaborate strategies to protect major organs, and the investment of numerous medical resources. Mitochondrial dysfunction, which acts as the center of energy metabolism, is clearly the basis of pathogenesis. Drugs that act on PGC1α, the master regulator of mitochondrial biosynthesis, have shown useful effects in the treatment of sepsis; therefore, we investigated the efficacy of ZLN005, a PGC1α agonist, and found significant improvement in overall survival in an animal model. The mode of action of this effect was examined, and it was shown that the respiratory capacity of mitochondria was enhanced immediately after administration and that the function of TFEB, a transcriptional regulator that promotes lysosome biosynthesis and mutually enhances PGC1α, was enhanced, as was the physical contact between mitochondria and lysosomes. ZLN005 strongly supported immune defense in early sepsis by increasing lysosome volume and acidity and enhancing cargo degradation, resulting in a significant reduction in bacterial load. ZLN005 rapidly acted on two organelles, mitochondria and lysosomes, against sepsis and interactively linked the two to improve the pathogenesis. This is the first demonstration that acidification of lysosomes by a small molecule is a mechanism of action in the therapeutic strategy for sepsis, which will have a significant impact on future drug discovery.

## Introduction

Sepsis is an immune dysfunction based on bacterial infection that affects 48.9 million people each year, claims 11 million human lives and is the cause of significant medical expenditures (1). Treatment in clinical practice includes infusions for fluid balance, control of the causative infection with antimicrobial agents, intervention for coagulation abnormalities, and support for the cardiorespiratory systems (2). Bacterial control, at its most upstream location, has been extremely lacking on the medical side due to the emergence of multidrug-resistant bacteria associated with high antimicrobial use (3) and a marked decrease in the speed of development of new antimicrobial agents that have become weasel words (4). In recent years, the importance of how to protect major organs from damage due to hyperimmune reactions in early sepsis and immunosuppression in the late phase, in addition to preserving microcirculation (2), has become clear, and great progress has occurred in intervening in innate immunity on the front line in the fight against bacteria. In addition, the development of biologics that suppress the overproduction of inflammatory cytokines has also been active (5). However, although therapeutic approaches for conditions caused by hyperimmune reactions are beginning to be enhanced, there has not been significant progress in strategies to promote bacterial clearance.

It has been shown in animal studies that in the initial response in sepsis, efficient bacterial clearance by phagocytes reduces the bacterial load on the organism, which in turn leads to improved survival (6). Phagocytes, which play a central role in innate immunity against bacteria, are mainly formed from monocytic macrophages and neutrophils, and their cellular metabolism has been shown to have a significant impact on functional regulation (7). In T cells, effector cells are involved in the glycolytic system, and memory cells are highly dependent on mitochondrial respiration (8). However, metabolic transformation is also heavily involved in the regulation of polarization in macrophages (9). Pathological conditions such as immunological exhaustion and senescence, which have been extensively studied in T cells (10, 11), have also been shown to occur in the monocyte-macrophage lineage, making them vulnerable to bacterial infection (12). If phagocytes follow the lead of lymphocytes, dysfunction may be caused by altered metabolism, and interventions to address them may have great potential benefit.

At the 3rd International Consensus Conference, sepsis was defined as organ failure due to a defective host response to infection (13), and mitochondrial dysfunction has been shown to underlie organ failure (14). Mitochondria generate energy and serve as the hub of innate immunity (15). Starting with the depolarization of mitochondrial membrane potential in peripheral blood demonstrated in clinical patient samples (16), impairment of mitochondrial complex I function in skeletal muscle cells was reported (17). In the peripheral blood of septic patients, a loss of function of mitochondrial complex V, ATP synthase/ATPase, has been reported from several centers (18, 19), and even a loss of function of mitochondrial complexes III and IV in the peripheral blood of patients has been recognized (20). In addition, this ATPase dysfunction correlated well with prognosis (19). Furthermore, a good correlation between increased mitochondrial biosynthesis or functional recovery during treatment and improved prognosis was reported (21, 22), and the presence of mitochondrial Sirt3 in the blood was shown to be a prognostic factor (23). We have demonstrated improved survival in an LPS-treated endotoxemia model by shifting the macrophage phenotype to M2-like by administering an eicosanoid analog that favors mitochondrial metabolism (24).These findings provide a foundation for treating sepsis by intervening in mitochondrial function.

Professional phagocytes and epithelial cells are at the forefront of processing bacteria and attempt to digest bacteria through three pathways after phagocytosis (25). When bacteria are opsonized by immunoglobulin or complement, they associate with Fcγ receptors, complement receptors, and other receptors present on the plasma membrane, leading to cytoskeletal reorganization and ruffle formation for uptake and phagolysosome formation (26). However, if the phagosome is disrupted by the evading effort of bacteria and they escape into the cytoplasm, xenophagy is triggered (27, 28). Toll-like receptors (TLRs), which exist as sensing receptors on the phagosome luminal side, signal Beclin-1, which is essential for autophagy initiation, followed by phagophore elongation. However, the damaged phagosome exposes galectin in the lumen to the cytoplasm, and the bacterial surface that has escaped from the phagosome is ubiquitinated, resulting in the binding of the phagophore’s sequestosome-1-like receptors (SLRs), such as sequestosome-1 (p62), optineurin, and NDP 52, which play a role as xenophagy receptors, to them, consequently recruiting microtubule-associated protein 1 light chain 3 (LC3). Subsequently, the phagophore undergoes autophagosome formation to reach lysosome fusion. As a third pathway, signals from TLRs and Dectin-1 in bacteria-encapsulated phagosomes induce the association of LC3, which is called LC3-associated phagocytosis (LAP) (29). This pathway promotes fusion with lysosomes earlier than conventional phagosome maturation (30). The process from phagocytosis to fusion with lysosomes provides an opportunity for intracellular parasitism of bacteria, which has been identified as a potential drug target (31).

As phagosome maturation progresses, the acidity of the lumen increases, accompanied by a transformation of the lipids of its component membranes (32). Ultimately, fusion with the most acidic lysosomes exposes pathogens such as bacteria and viruses to a process of denaturation and digestion by up to 60 different hydrolytic enzymes that are optimally suited to highly acidic environments (33). Hydrogen ion enrichment, which is 500 times higher than the cytosolic concentration, is maintained by vacuolar H^+^-ATPase (V-ATPase) pumping hydrogen ions into the lumen and by several antiporter ion channels (33, 34). The V-ATPase that gives rise to lysosomal acidity is composed of a peripheral membrane complex V1 with three catalytic sites as ATPases and an integral membrane complex V0 with proton pores (35). The structure of V-ATPase is well conserved from S. cerevisiae to mammals, and the regulation is mediated by reversible V0-V1 disassembly (36–38). In sepsis, lysosome biogenesis in the macrophage-monocyte lineage is enhanced (39) but may not be able to ensure sufficient acidity to kill all bacteria. V-ATPase dysfunction has been shown to have a major impact on the pathogenesis of neurodegenerative diseases (40). V-ATPase dysfunction is predicted to play a major role not only in neurodegenerative diseases but also in a variety of pathological conditions, including sepsis, which has not been recognized until now (34).

Intervention in mitochondrial function is a strategy with great potential to induce phenotypic alterations in immune cells. ZLN005 has shown various benefits as a PGC1α agonist through the promotion of mitochondrial biosynthesis. We investigated the effect on a polymicrobial sepsis model and the mode of action of ZLN005 in this study. Focusing on the association with the lysosome, a TFEB target that PGC1α mutually enhances, we investigated the molecular mechanism of ZLN005 in macrophages and monocytes, which are the first line of defense in sepsis and determine its outcome.

## Materials and methods

### Sepsis model mice

C57BL/6 mice were purchased from Shimizu Laboratory Supplies Co., Ltd. (Kyoto, Japan). Mice were housed in specific-pathogen-free conditions with free access to food and water. Mice were anesthetized by inhalation of isoflurane (099-06571, FUJIFILM Wako Pure Chemical Corporation, Tokyo, Japan). A midline incision was made, followed by externalization, and the cecum was then ligated at half the distance between the distal pole and the base of the cecum and punctured with a 21-gauge needle (NN-2116R, Terumo Corporation, Tokyo, Japan). Next, a small amount of fecal mass from the punctured cecum was gently squeezed out to ensure patency of punctures, the cecum was relocated, and 6/0 Ethicon PROLENE Suture (Ethicon, Inc., Raritan, NJ, USA) was used to close the peritoneum and skin. Sham-operated mice underwent only incision and cecum exteriorization.

### Mice intraperitoneal injection of ZLN005

ZLN005 (S7447, Selleck Chemicals, Houston, TX, USA) stock solutions were prepared in dimethyl sulfoxide (DMSO) (046-21981, FUJIFILM Wako Pure Chemical Corporation) adjusted to a 10 mM concentration. Mice were injected with ZLN005 (12 mg/kg) or the same amount of DMSO as the control group every day from Day 0 to Day 2. Mice were examined continuously for survival until 6 days post-CLP.

### Isolation of mouse peritoneal cavity cells

The outer skin of the peritoneum was cut with scissors and gently pulled back to expose the inner skin lining the peritoneal cavity. The inner skin was punctured with a 18G Surflo I.V. Catheter (SR-FS1851, Terumo Corporation, Tokyo), and 4 ml of ice-cold PBS was injected. After injection, the abdomen was gently massaged to dislodge peritoneal cavity cells. The collected fluid was filtered through a Falcon 40-µm Cell Strainer (352340, Corning Inc., Corning, NY, USA) and centrifuged at 800 × g for 5 minutes. Cell pellets were resuspended in 5 ml of 1× Lysing Buffer (555899, Becton, Dickinson and Company, Franklin Lakes, NJ, USA) and incubated for 5 minutes at room temperature to lyse erythrocytes. Then, 10 ml of PBS was added and centrifuged at 800 × g for 5 minutes, the supernatant was discarded, and the cells were resuspended in PBS or culture medium.

### Population analysis of peritoneal cavity cells

We harvested peritoneal cavity cells 24 hours after CLP. The cells were stained with a PE anti-mouse/human CD11b antibody (101207, BioLegend, Inc., San Diego, California, USA) and FITC anti-mouse F4/80 antibody (123108, BioLegend, Inc.) or PE Rat IgG2b, κ isotype ctrl antibody (400608, BioLegend, Inc.) and FITC Rat IgG2a, κ isotype ctrl antibody (400505, BioLegend, Inc.) for 30 minutes at 4°C after blocking the nonspecific Fc receptor using the Fc blocking reagent (130-059-901, Miltenyi Biotec, Bergisch Gladbach, Germany) for 10 minutes at room temperature. After staining, the cells were washed immediately and resuspended in AutoMACS Runnig Buffer (130-091-221, Miltenyi Biotec). Fluorescence data were collected using a SH800 (Sony Biotechnology Inc., Tokyo, Japan). The flow cytometry files were analyzed using FlowJo software (Ver. 10.8.1, Becton, Dickinson and Company).

### Echocardiographic measurement

Chest hair was removed with cream 1 day before echocardiography was performed using VisualSonics Vevo 2100 equipped with an 18- to 38-MHz probe (VisualSonics, Toronto, ON, Canada). Mice were anesthetized by inhalation of isoflurane at 24 hours post-CLP. The left ventricle was assessed in the parasternal short-axis view. Left ventricular end-systole or end-diastole was defined as the period when the left ventricular lumen was shortest or most dilated, respectively. Diastolic left ventricular (LV) internal diameters, systolic LV internal diameters, diastolic LV anterior wall, and diastolic LV posterior wall were measured from the LV M-mode tracing at the papillary muscle level. LV wall thickness was calculated as the average of anterior and posterior wall thicknesses.

### Histology and inflammatory scores

The heart, lung, liver, right kidney, and spleen of mice were collected 24 hours post-CLP and fixed with 4% paraformaldehyde (163-20145, FUJIFILM Wako Pure Chemical Corporation). All tissues were embedded in paraffin, cut into sections, and stained with hematoxylin and eosin (HE). Liver inflammatory scores were assessed based on the severity of necrosis, bleeding, and infiltration in the liver using the method described in a previous report (24). Lung inflammatory scores were assessed based on the severity of edema, intra-alveolar cell infiltration, congestion and alveolar hemorrhage using the method described in a previous report (41).

### Cell culture

THP-1 cells of a human monocytic leukemia cell line were cultured in Roswell Park Memorial Institute 1640 medium (11875-093, RPMI1640, Thermo Fisher Scientific Inc., Waltham, MA, USA) supplemented with 10% fetal bovine serum (FBS, 10270-106, Thermo Fisher Scientific Inc.). Cells were incubated at 37°C in a humidified 5% CO_2_ incubator. For the THP-1 inflammation model, the cells were seeded in 12-well cell culture plates (353043, Corning Inc.) at a density of 5 × 10^5^ cells per well in growth medium containing 10 nM phorbol 12-myristate 13-acetate (PMA, AG-CN2-0010-M001, Adipogen Life Sciences Inc., San Diego, CA, USA). After 48 hours, the supernatant of the medium was carefully removed to avoid detaching the cells attached to the bottom of the plate and replaced with new medium containing 1 µM ZLN005 or DMSO (0.1%) as a control. Forty-eight hours later, 1 µg/ml LPS (lipopolysaccharides, 125-05181, FUJIFILM Wako Pure Chemical Corporation) was added, and the cells were harvested at each time point and used for experiments.

For PI3K inhibition, the cells were seeded in the plate and added 200 nM Wortmannin (AG-CN2-0023-M001, Adipogen Life Sciences Inc.). For PGC1α gene knock down, cells were nucleofected with 10 nmole siRNA-PGC1α (4427037, Thermo Fisher Scientific Inc.) and 10 nmole siRNA- negative control (4390844, Thermo Fisher Scientific Inc.) using the Nucleofector 2b (Lonza, Walkersvill, MD, USA) according to the manufacturer’s protocol. For autophagy flux analysis, cells were stimulated with 1 µg/ml LPS for 6 hours, followed by 50nM bafilomycin A1 (B0025, LKT Laboratories, Inc., Saint Paul, MN, USA) or 30nM chloroquine (08660-04, Nacalai Tesque Inc., Kyoto, Japan) for 2 or 6 hours.

### RNA isolation, reverse transcription PCR and quantitative PCR

Total RNA from cells and tissues was extracted using TRIzol (15596018, Thermo Fisher Scientific Inc.) and a Direct-zol RNA MiniPrep Kit (R2052, Zymo Research, Irvine, CA, USA) with DNase I, according to the manufacturer’s recommendations. To perform the qRT−PCR assay, 100 ng of total RNA was reverse-transcribed using the PrimeScript RT Reagent Kit (RR036A, Takara Bio, Shiga, Japan) and a T100 thermal cycler (Bio-Rad Laboratories, Inc.). qRT−PCR was performed with Kapa SYBR Fast qPCR Kit Master Mix (2×) Universal (KK4602, Kapa Biosystems Ltd., Wilmington, MA, USA) on a CFX connect real-time system (Bio-Rad Laboratories, Inc.). The relative gene expression levels were normalized to *GAPDH* (or *Gapdh*) expression. The mtDNA copy number (CN) was estimated from the content ratio of 12S rRNA on mtDNA and *ACTB* (or *Actb*) on nuclear DNA by delta cycle threshold-based relative quantification.

### Mitochondrial membrane potential (Δφ)

Cells were resuspended at a density of 1 × 10^5^/ml in culture medium containing 100 nM MitoTracker Green FM (MitoG, Thermo, M7514, Fisher Scientific, Inc.) and 100 nM Image-iT TMRM Reagent (TMRM, T668, Thermo Fisher Scientific, Inc.) and incubated at 37°C for 30 minutes. After staining, the cells were washed immediately, resuspended in AutoMACS running buffer, and evaluated using an SH800. The fluorescence intensity was analyzed by FlowJo, and the numeric value was calculated by dividing the fluorescence intensity of TMRM by the fluorescence intensity of MitoG (TMRM/MitoG) as an index of Δφ.

### Measurement of mitochondrial reactive oxygen species levels

Cells were resuspended at a density of 1 × 10^5^/ml in culture medium containing 5 μM MitoSOX Red mitochondrial superoxide indicator (MitoSOX, M36008, Thermo Fisher Scientific, Inc.) and incubated at 37°C for 30 minutes. After staining, the cells were washed immediately, resuspended in AutoMACS running buffer, and evaluated using an MA900 (Sony Biotechnology Inc., Tokyo, Japan).

### Measurement of cellular reactive oxygen species

Cells were resuspended at a density of 1 × 10^5^/ml in culture medium containing 5 μM CellROX™ Deep Red (CellROX, C10491, Thermo Fisher Scientific, Inc.) and incubated at 37°C for 30 minutes. After staining, the cells were washed immediately, resuspended in AutoMACS running buffer, and evaluated using an SH800.

### Mitophagy detection assay

To detect mitophagy, the pMX retroviral vector carrying Monomeric Keima Red (mKeima Red) was transfected into THP-1 cells. The mKeima Red-expressing cells were selected using SH800 1 week after retrovirus transfection. We performed one more sorting to obtain over 95% cells expressing mKeima Red. The acidic mKeima Red signal was detected by an Attune NxT Flow Cytometer (Thermo Fisher Scientific). mKeima Red was set at 488-nm (pH 7) and 561-nm (<pH 6) lasers with 590/40-nm and 615/20-nm emission filters, respectively. We defined the mitophagy index as the ratio of acidic (<pH 6) mKeima Red signal-positive cells to DMSO control cells at 0 hours (42).

### Phagocytosis assay

Phagocytosis was assessed using a Phagocytosis Assay Kit IgG-FITC (500290, Cayman Chemical, Ann Arbor, MI, USA). Cells were suspended at a concentration of 3 × 10^5^ in 1 ml of culture medium and stained with the Latex Beads-rabbit IgG-FITC Complex from the kit for 20 minutes at 37°C. After staining, the cells were centrifuged at 400 × g for 5 minutes and resuspended in 200 μl of autoMACS running buffer, and evaluated using an SH800.

### Lysosomal acidification

Lysosomal acidification was assessed using pHrodo Green dextran (P35368, Thermo Fisher Scientific, Inc.). Peritoneal cavity cells suspended at a concentration of 3 × 10^5^ in 1 ml of culture medium were stained with 50 μg/ml pHrodo Green dextran from the kit for 20 minutes at 37°C. After staining, the cells were centrifuged at 400 × g for 5 minutes and resuspended in 200 μl of autoMACS running buffer, and evaluated using an SH800.

### Lysosomal staining

Lysosomes were stained with LysoTracker Red DND-99 (LysoTracker Red, L7528, Thermo Fisher Scientific, Inc.). Cells were suspended at a concentration of 3 × 10^5^ in 1 ml of culture medium and stained with 50 nM LysoTracker Red from the kit for 15 minutes at 37°C. After staining, the cells were centrifuged at 400 × g for 5 minutes and resuspended in 200 μl of autoMACS running buffer, and evaluated using an SH800.

### Lysosomal proteolysis

Cells were incubated with 0.1 mg/ml DQ Green BSA (DQ BSA, D12050, Thermo Fisher Scientific, Inc.) for 4 hours, washed two times and incubated for 3 hours in fresh media to allow lysosomal accumulation of DQ BSA. Lysosomes were labeled with 50 nM Lysotracker Red for 15 minutes. After staining, the cells were centrifuged at 400 × g for 5 minutes and resuspended in 200 μl of autoMACS running buffer, and evaluated using an SH800.

### Measurements of respiratory function and glycolysis

An XFe96 extracellular flux analyzer (Agilent Technologies, Santa Clara, CA, USA) was used to measure cellular respiratory function. Cells were suspended in Seahorse XF RPMI medium (Agilent Technologies) containing 10 mM glucose, 1 mM pyruvate, and 2 mM L-glutamine and seeded on XFe96-well microplates (101085-004, Agilent Technologies) coated with Cell-Tak (CLS354240, Corning Inc.) at a density of 1 × 10^5^ cells per well. After seeding, the cells were equilibrated in a non-CO_2_ incubator for 20 minutes and used in the assay. For measurement of respiratory function, oligomycin (2 μM), carbonyl cyanide p-trifluoromethoxyphenyl hydrazone (FCCP, 2 μM) and rotenone/antimycin A (0.5 μM), which were adjusted using the reagents in the Seahorse XF Cell Mito Stress Test Kit (103015-100, Agilent Technologies), were sequentially added to each well after baseline measurement. The data are presented as the oxygen consumption rate (OCR; pmol/minute). Basal respiration, ATP production, maximal respiration, proton leakage, spare respiratory capacity, nonmitochondrial oxygen (non-MTC) and coupling efficiency were calculated using Wave Controller 2.4 (Agilent Technologies).

For the measurement of glycolysis, glucose (10 mM), oligomycin (1 μM) and 2-deoxy-D-glucose (2-DG, 50 mM), which were adjusted using the reagents in the Seahorse XF cell glycolysis stress test kit (103020-100, Agilent Technologies), were sequentially added to each well after baseline measurement. The data are presented as the extracellular acidification rate (ECAR; mpH/minute). Glycolysis, glycolytic capacity and glycolytic reserve were calculated using Wave Controller 2.4.

### Peritoneal bacterial quantification

The mice were euthanized with isoflurane at 24 hours post-CLP. The skin of the abdomen was cut open after disinfection, and without damage to the muscle layer, the peritoneal cavity was washed with 4 ml of sterile PBS with 2 mM EDTA. The obtained peritoneal lavage was diluted 1:10,000 in PBS, and 40 µl of the diluted solution was plated on LB agar (22700-025, Thermo Fisher Scientific, Inc.) without any antibiotics. After incubation in a nonhumidified incubator at 37°C for 24 hours, the plates were photographed by a ChemiDoc Imaging System, and colony-forming units (CFUs) were counted in ImageJ (Version 1.53t, National Institutes of Health, Bethesda, MD, USA). The results are expressed as the number of CFUs per cm^2^.

### Subcellular isolation for Western blotting

THP-1 cells were resuspended in fractionation buffer (20 mM HEPES, 10 mM KCl, 2 mM MgCl_2_, 1 mM EDTA, 1 mM EGTA, 1 M DTT, 1/100 Protease Inhibitor Cocktail Set I (FUJIFILM Wako Pure Chemical Corporation), pH 7.2) and homogenized by passing twenty times through a 29-gauge needle. The lysate was maintained on ice for 20 minutes and then was separated into pellet containing nuclei and supernatant containing cytoplasm, membrane and mitochondria by centrifuging at 720 g for 5 minutes. The supernatant was centrifuged again at 12,000 g for 10 minutes. The cytoplasmic supernatant from the pellet was transferred to a clean tube. Nuclear pellet was washed with 500μL of fractionation buffer and centrifuged again at 720 g for 10 minutes. The pellet in fractionation buffer was resuspended, then sonicateded to shear genomic DNA and homogenize the lysate. These proteins were analyzed by Western blotting.

### Western blotting

Cytoplasmic protein was dissolved in RIPA buffer (182-02451, FUJIFILM Wako Pure Chemical Corporation), boiled for 10 minutes, electrophoresed through 10% Mini-PROTEAN TGX Precast Protein Gels (4561036, Bio-Rad Laboratories Inc.) and electroblotted onto a PVDF transfer membrane (IPVH00010, Merck KGaA, Darmstadt, Germany). The membrane was blocked with PBS containing 5% skim milk and 0.05% Tween 20 (P1379, Merck KGaA) and incubated for 1 hour with a PGC1a (sc-517380, Santa Cruz Biotechnology, Dallas, TX, USA), TFEB (ab267351, Abcam plc., Cambridge, UK), Akt (9272, Cell Signaling Technology, Inc., Danvers, MA, USA), phospho-Akt (Ser473) (9271, Cell Signaling Technology, Inc.), PI3 kinase p85 (19H8) (4257, Cell Signaling Technology, Inc.), phospho PI3 kinase p85 (Tyr458)/p55 (Tyr199) (4228, Cell Signaling Technology, Inc.), S6 ribosomal protein (5G10) (2217, Cell Signaling Technology, Inc.), phospho-S6 ribosomal protein (Ser235/236) (2211, Cell Signaling Technology, Inc.), AMPKα (23A3) (2603, Cell Signaling Technology, Inc.), phospho-AMPKα (Thr172) (40H9) (2535, Cell Signaling Technology, Inc.), LC3 (0231-100BIOTIN/LC3-5F10, nanotools GmbH, Teningen, Germany), GAPDH (MAB374, Merck KGaA), and α-tubulin (66031-1-Ig, Proteintech Group, Inc., Rosemont). After washing, the membrane was incubated with a 1:5000 dilution of anti-mouse IgG (7076S, Cell Signaling Technology, Inc.) or anti-rabbit IgG HRP-linked antibody (7074S, Cell Signaling Technology, Inc.) in blocking buffer. Subsequently, the blots were developed using an Clarity Western ECL Substrate (1705060, Bio-Rad Laboratories Inc.) or Clarity Max Western ECL Substrate (1705062, Bio-Rad Laboratories Inc.), and the protein bands were visualized using a VersaDoc or ChemiDoc Imaging System (Bio-Rad Laboratories Inc.). Protein levels were quantified using ImageJ.

### Immunocytochemistry

Cells were fixed in 4% paraformaldehyde at 4°C for 15 min. in the presence of a protein-blocking solution consisting of PBS supplemented with 5% normal goat serum (X090710-8, Agilent Technologies Inc., SantaClara, CA, USA). The cells were incubated overnight with anti-TFEB antibody (ab267351, Abcam plc.) in PBS at 4°C. The cells were washed extensively in PBS and incubated at room temperature for 30 min with a anti-rabbit IgG (H + L) antibody tagged with Alexa FluorTM 488 (Thermo Fisher Scientific, Inc.). The nuclei were counterstained with 4′,6-diamidino-2-phenylindole (DAPI; diluted 1:500, #5748, FUJIFILM Wako Pure Chemical) in PBS at room temperature for 30min. We obtained the fluorescence images using a Biorevo BZ-9000 fluorescence microscope (Keyence Corporation, Osaka, Japan). The identification of TFEB migrated to nuclear was performed using ImageJ. First, the multicolor image was separated into TFEB- and DAPI-stained images. These images were converted to binarized images by thresholding, where a foreground pixel was assigned the maximum value of 255 and background pixels were assigned the minimum possible value of 80. The area where the TFEB and DAPI areas overlap is defined as the nuclear TFEB. The percentage of cells in the image with nuclear TFEB was calculated.

### Mitochondrial network analysis

We performed mitochondrial morphology analysis by using the Mitochondrial Network Analysis (MiNA) toolset, which was downloaded from https://github.com/stuartlab (43). To obtain precise results, we first improved the quality of the images. Choices for image preprocessing, including an unsharp mask and enhanced local contrast, are presented to the user through the MiNA interface. For analysis, the image was first binarized by thresholding, assigning a maximum value of 255 to foreground pixels and a minimum value of 0 to background pixels. Next, using ImageJ’s built-in skeletonization function, the binary image was converted into a skeleton that represents the features of the original image as a wireframe of one-pixel-wide lines. All pixels within a skeleton were then grouped into three categories: end point pixels, slab pixels, and junction pixels.

We evaluated the area of the mitochondrial footprint, slab pixels and junction pixels of individual cells. The mitochondrial footprint is the number of pixels in the binary image containing signal multiplied by the area of a pixel if the calibration information is present.

### Mitochondria lysosome contact site analysis

Analysis was performed using ImageJ. First, the multicolor image was separated into images stained with MitoTracker Green and LysoTracker Red. These images were converted to binarized images by thresholding, where a foreground pixel was assigned the maximum value of 255 and background pixels were assigned the minimum possible value of 100. The contact site is the area where the regions of interest (ROIs) of the mitochondria and lysosomes overlap. We evaluated the ratio of contact sites to the mitochondrial area of individual cells.

### Statistical analysis

The results are presented as the means ± standard deviations. The statistical significance of differences among groups was evaluated using parametric unpaired t tests for bar graphs. Mantel−Cox tests were used for statistical analysis of datasets of Kaplan−Meier survival curves (Prism 9 software, GraphPad Prism Software Inc., San Diego, CA, USA). *P* < 0.05 was considered to indicate significance.

## Results

### Improved survival and anti-inflammatory effects of ZLN005 in the CLP sepsis model

**Figure 1:**
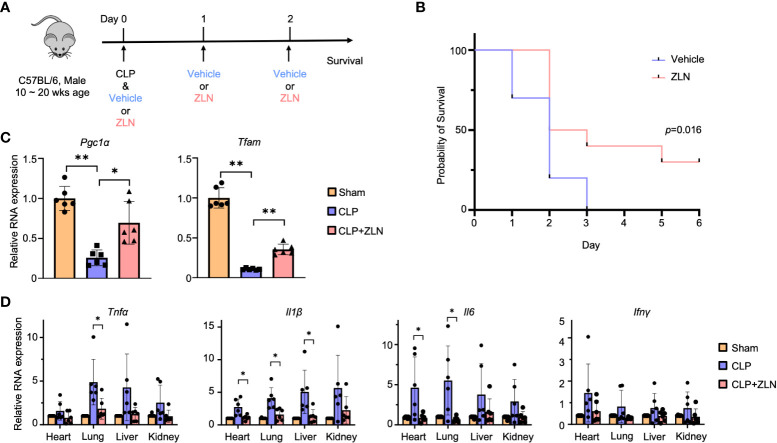
**(A)** C57BL/6 mice were treated intraperitoneally with Vehicle or ZLN005 every 24 hours post-cecal ligation and puncture (CLP). **(B)** Kaplan–Meier survival curve of mice post-CLP (n = 9). **(C)** The mRNA expression of *Pgc1α* & *Tfam* at 24 hours post-CLP (n = 6). **(D)** The mRNA expression of proinflammatory cytokines at 24 hours post-CLP (n = 6). P values ** < 0.0001, P values *0.0001 to 0.05.

Cecal ligation perforation (CLP) has been shown to be the most appropriate animal model for polymicrobial sepsis and was used in this study. The severity of CLP can be controlled by the location of the ligation and the number of perforations (**Supplementary Figure 1A**). The ligation was performed at the base or middle of the cecum, and the severity was controlled by performing one or two perforations with a 20 Gauge needle beyond the ligature. A model in which the base of the cecum is ligated and two perforations are made has a 50% survival rate on postoperative Day 2, and this method will be used for future studies (**Supplementary Figure 1B**). ZLN005, the efficacy of which was verified in this study, is being developed for clinical application as a drug that acts as a PGC1α activator (**Supplementary Figure 1C**). The effect of ZLN005 on naïve THP-1 was observed at the mRNA and protein levels over time, with a 2-fold increase in expression at 48 and 36 hours, respectively (**Supplementary Figure 1D**). To examine the therapeutic effect, the scheme was to inject ZLN005 intraperitoneally for 3 consecutive days after CLP was performed in 10-20-week-old C57BL/6 mice (**Figure 1A**).

In the sham surgery group, 25% of the mice died after 24 hours, while there were no deaths in the ZLN005 group. At 72 hours, all sham-operated mice were dead; however, 40% of the ZLN005-treated mice were alive, and 30% remained alive after 6 days, with Kaplan−Meier survival analysis results revealing significantly better survival in the ZLN005 group (**Figure 1B**). In addition, the behavior of the animals just 2 hours after the first drug administration after CLP was highly contrasting. In the sham-operated group, the mice remained in one place, were very unresponsive to stimuli and rarely moved (**Supplementary Movie 1**). In the ZLN005 group, however, mice spontaneously moved around and rarely stayed in one place, and they rapidly exhibited escape behaviors in response to stimuli (**Supplementary Movie 2**). These early behavioral changes suggest an immediate effect of ZLN005 on bacteria leaking into the peritoneal cavity in addition to its mechanism of action as a PGC1α activator at the transcriptional level.

In the CLP model, the intraperitoneal macrophage-monocyte lineage has been reported to exhibit a decisive response in early pathogenesis (44), so we examined the expression of *Pgc1α*, for which ZLN005 has been reported, and *Tfam*, which plays an essential role in mitochondrial biogenesis, in intraperitoneal cells, including macrophages and monocytes. Both transcripts were significantly elevated (**Figure 1C**). In the CLP model, inflammatory cytokines were drastically increased from the early stage, and excess inflammatory cytokines played an important role in pathogenesis (**Figure 1D**). *Tnfα* and *Il1β* were most highly expressed in the liver, followed by the kidney, lung, and heart in the sham group. *Il6* was most highly expressed in the kidney in the sham group but was significantly downregulated by ZLN005 treatment, as were *Tnfα* and *Il1β*, to a level similar to that in the sham group. Although *Infγ* expression itself was lower than that of other cytokines, the inhibitory effect of ZLN005 was significant in the heart and lung, down to the expression level of the sham group. These results indicate a high anti-inflammatory effect of ZLN005.

### Organ protection of ZLN005 in the CLP sepsis model

**Figure 2:**
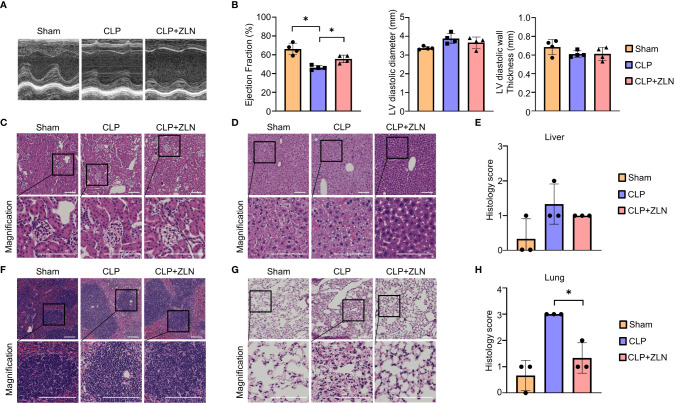
**(A)** Representative M–mode ultrasound cardiography and percentages of ejection fraction **(B)**, left ventricular diameter and left ventricular diastolic wall thickness at 24 hours post-CLP (n = 4). **(C, D, F, G)** Representative examples of hematoxylin-eosin staining of the kidney **(C)**, liver **(D)**, spleen **(F)**, and lung **(G)** of mice at 24 hours post-CLP. **(E)** Histological score of the liver in mice at 24 hours post-CLP (n = 3). **(H)** Histological score of the lung in mice at 24 hours post-CLP (n = 3). All bars in the images are 100 μm. P values *0.0001 to 0.05.

Next, we examined how the anti-inflammatory effects of ZLN005 affect the major organs. The early surge of various inflammatory cytokines in the CLP model, also called the cytokine storm, causes circulatory collapse and hypercoagulability, leading to cell death of immune system cells. Twenty-four hours after CLP, ultrasound cardiography was performed (**Figure 2A**). The sham group showed an ejection fraction from the 60% to 40% range, while in the group treated with ZLN005, the ejection fraction remained in the 50% range (**Figure 2B**). Cardiac pathological examination revealed no significant findings, including cellular infiltration or hemorrhage, suggesting that dysfunction could be caused by fluid factors such as proinflammatory cytokines and coagulation factors and that the suppression of cytokine storms by ZLN005 could work to preserve cardiac function (**Supplementary Figure 2**). The kidneys, like the heart, showed no significant changes (**Figure 2C**). The liver, however, showed a disorganized lobular structure, hemorrhagic lesions, and cellular infiltration in the sham group and slightly improved findings in the group treated with ZLN005 (**Figure 2D**), but quantification failed to reveal any significant changes (**Figure 2E**). The spleen showed a large disruption of follicular structure in the sham group, while the follicular structure was well preserved in the group treated with ZLN005 (**Figure 2F**). The lungs showed the most pronounced pathology, with cellular infiltration clearly suppressed in the ZLN005 group, the interstitium preserved, and edema very mild (**Figure 2G**). Quantification also showed significantly less damage in the ZLN005 group (**Figure 2H**).

**Figure 3:**
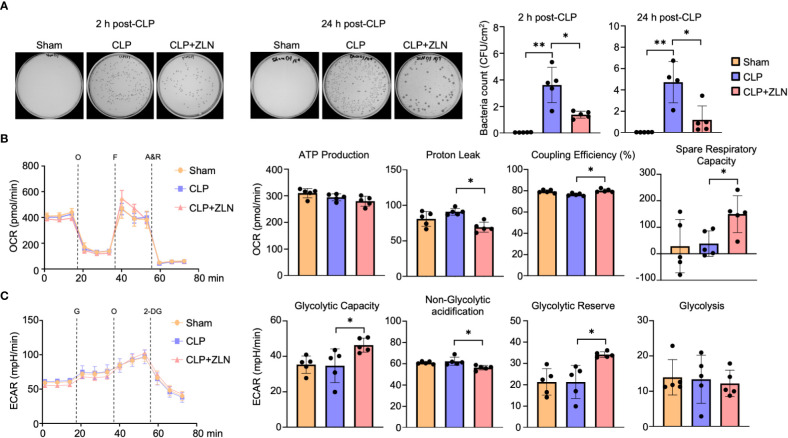
**(A)** Representative images of colony-forming units (CFU) and measurement of bacterial colony number in peritoneal lavages at 2 or 24 hours post-CLP. (B: Sham: n = 4, CLP: n =4, CLP + ZLN005: n = 4, C: Sham: n = 5, CLP: n =4, CLP + ZLN005: n = 5). **(B, C)** Changes in the OCR **(B)** and ECAR **(C)** of THP-1 cells in the inflammation model, as measured using flux analysis. O, oligomycin; F, FCCP; A&R, antimycin and rotenone; G, glucose; 2-DG, 2-deoxy-D-glucose (n = 5). Each index of glycolysis was calculated from the ECAR measurement results. Each index of mitochondrial respiratory function was calculated from the OCR, glycolysis was calculated from the ECAR measurement results. P values ** < 0.0001, P values *0.0001 to 0.05.

We investigated the effect of ZLN005 on the elimination of intraperitoneal bacteria in the CLP model. Ascites was collected 2 and 24 hours after CLP treatment, and colonies were measured by bacterial culture. At 2 hours, the number of colonies had already decreased by more than half in the ZLN005-treated group, and by 24 hours, the number of colonies had further decreased by about one-fourth (**Figure 3A**). This indicates that ZLN005 promotes the elimination of bacteria released into the peritoneal cavity. These results suggest that ZLN005 was able to make the intraperitoneal monocyte-macrophage lineage more controllable against an uncontrollable bacterial load. The early onset of action at 2 hours suggests that the mechanism of action is directly related to metabolism and digestion rather than requiring a process of transcription and translation.

### Mitochondrial functional alterations by ZLN005

In the CLP model, the mitochondrial respiratory capacity and metabolic capacity of the glycolytic system in intraperitoneal cells after 1 day of treatment were examined.Intraperitoneal cells after CLP treatment were harvested and profiles of mitochondrial oxidative phosphorylation (OXPHOS) and glycolytic capacity were obtained by sequential use of respiratory chain complex inhibitors and glycolytic system inhibitors using Seahorse. Intraperitoneal cells on postoperative day 1 did not show significant changes in OXPHOS with CLP, but administration of ZLN005 decreased proton leak, resulting in a significant increase in coupling efficiency. In addition, spare respiration capacity was significantly increased (**Figure 3B**). On the other hand, in the glycolytic system, as in OXPHOS, CLP treatment did not cause significant changes on the first postoperative day, but administration of ZLN005 significantly increased glycolytic capacity and glycolytic reserves (**Figure 3C**). This indicates that ZLN005 is responsible for the improvement in reserve capacity of both metabolic pathways.

**Figure 4:**
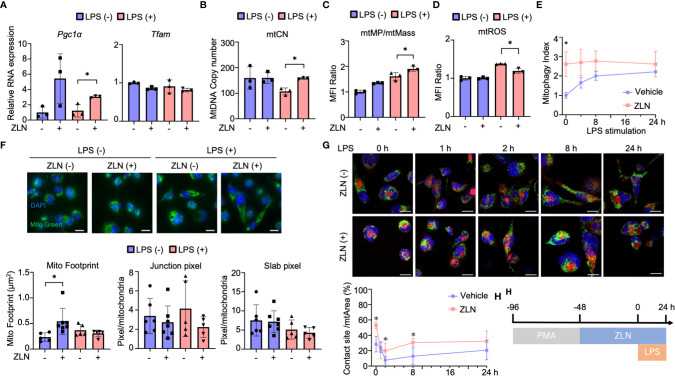
**(A)** The mRNA expression of *PGC1α* and *TFAM* in THP-1 cells in the inflammation model at 24 hours post-LPS administration (n = 3). **(B)** The absolute copy number of THP-1 in the inflammation model at 24 hours post-LPS administration (n = 3). **(C)** MFI ratio of mtROS levels in THP-1 cells in the inflammation model at 24 hours post-LPS administration (n = 3). **(D)** MFI ratio of acidic mKeima Red signaling in THP-1 cells in the inflammation model at 0, 4, 8 and 24 hours post-LPS administration (n = 3). **(E)** MFI ratio of Δφ of THP-1 cells in the inflammation model at 24 hours post-LPS administration (n = 3). **(F)** Mitochondrial morphology of THP-1 cells in the inflammation model at 24 hours post-LPS administration. All bars in the images are 20 μm. The graph shows the mitochondrial footprint, junction pixel and slab pixel areas per cell (n=5). **(G)** Assessment of the mitochondria-lysosome contact site of THP-1 cells in the inflammation model at 0, 1, 2, 8 and 24 hours after LPS administration. **(H)** Protocol of the assessment of the mitochondria-lysosome tethering of in THP-1 cells. All bars in the images are 20 μm. The graph shows the ratio of the contact site area to the total mitochondrial area per cell (n = 5). P values *0.0001 to 0.05.

The mechanism of action of ZLN005 to date has been to enhance PGC1α at the transcriptional level, but there were no significant changes in the OXPHOS profile other than reserve capacity, and no alterations leading to a dramatic improvement in sterilization of the abdominal cavity. Therefore, we examined the mitochondrial function of ZLN005 using THP-1, a human macrophage strain, in order to dissect alternative mode of action of ZLN005 in this model. We examined the effect of ZLN005 on THP-1 in the model of stimulation with LPS at 24 hours after stimulation (**Supplementary Figure 3A**). *Pgc1α* mRNA was significantly elevated at the transcriptional level with or without LPS stimulation by ZLN005, whereas *Tfam* mRNA showed no significant change in either group (**Figure 4A**). However, mtDNA copy number was decreased by LPS stimulation but was significantly restored to that of the untreated group by treatment with ZLN005, regardless with ZLN005 (**Figure 4A**). PGC1α is a major regulator of mitochondrial biogenesis, and we examined mitochondrial DNA copy number as a phenotype. A significant increase in copy number was observed with ZLN005 (**Figure 4B**). Mitochondrial mass was measured by MitoGreen and showed no significant changes with LPS stimulation or ZLN005 administration (**Supplementary Figure 3B**). Mitochondrial membrane potential (mtMP) by TMRM (**Supplementary Figure 3C**) was corrected by mitochondrial mass, and mtMP/mtMass was increased by LPS stimulation and further increased by ZLN005 administration (**Figure 4C**). Mitochondrial ROS was significantly increased by LPS stimulation, but the increase was reversed by ZLN005 (**Figure 4D**, **Supplementary Figures 3D, E**). To measure mitophagy, we generated THP-1 cells, which constantly express MitoKeima Red (**Supplementary Figures 4A-D**), and examined the effect of ZLN005 under LPS stimulation over time. A slight induction of mitophagy was detected as early as 2 hours after ZLN005 administration, and no increase in mitophagy was recognized over time (**Figure 4E**). This enhanced mtDNA replication and increased mitophagy suggest enhanced mitochondrial turnover, but only to a small extent. Mitochondrial turnover might not contribute significantly to cellular phenotypic changes.

Because ZLN005 significantly affects mitochondrial biogenesis and turnover under LPS-stimulated conditions, we examined its effect on mitochondrial dynamics using THP-1 cells. Mitochondria were stained with TMRM to quantify mitochondrial morphology using ImageJ (**Supplementary Figure 5A**). Footprints indicating mitochondrial mass were more sensitive than those from MFI of MitoGreen by FACS (**Supplementary Figure 5B**), and ZLN005 was slightly increased in the unstimulated state but showed no change in the LPS-stimulated state (**Figure 4F**). The four groups showed no change in Slub pixel and Junction pixel, which are indicators of branching status (**Figure 4F**). We assessed the proximity of the two organelles with dye co-staining using dyes that stain mitochondria and lysosomes. We selected the cross-section with the largest lysosomal staining area and calculated the co-staining area at that cross-section. Although only one cross-section was evaluated, a clear increase in co-staining area was observed with exposure to ZLN005 throughout 24 hours following LPS exposure. These findings suggest tethering of the two organelles and suggest that lysosomes may be involved in another mechanism of action of ZLN005 (**Figure 4G, H**, **Supplementary Figure 5C**).

### Lysosomal alterations by ZLN005

**Figure 5:**
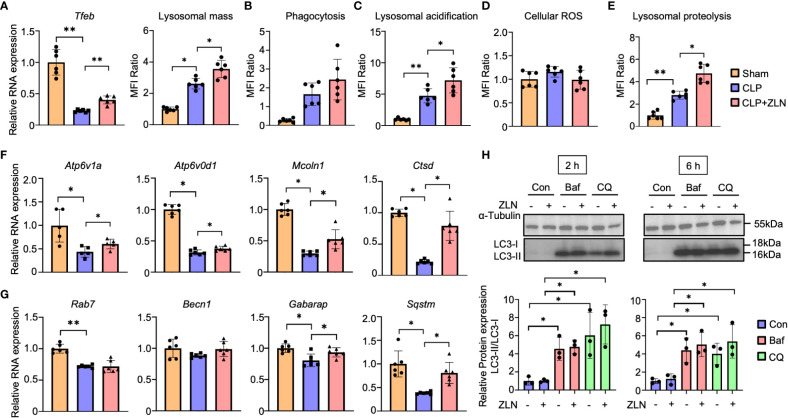
**(A)** The mRNA expression of *Tfeb* and MFI ratio of lysosomal staining of peritoneal cavity cells at 24 hours post-CLP (n = 6). **(B - D)** The assessment of phagocytosis (n = 6) **(B)**, lysosomal acidification **(C)**, and cellular ROS **(D)** in peritoneal cavity cells at 24 hours post-CLP. **(E)** Degradation of lysosomally preloaded DQ BSA in peritoneal cavity cells at 24 hours post-CLP. The graph shows the MFI ratio of degraded DQ Green BSA and LysoTracker Red. **(F, G)** The mRNA expression of lysosomal hydrolase **(F)** and autophagy-related genes **(G)** at 24 hours post-CLP (n = 6). **(H)** Western blotting analysis of the autophagy flux in THP-1 cells after 6 hours of exposure to LPS followed by 2 or 6 hours of treatment with the autophagy inhibitor. The graph shows the expression ratio of LC3-II protein corrected by LC3-I protein expression level (n = 3). (Con, Control; Baf, Bafilomycin A1; CQ, Chloroquine). P values ** < 0.0001, P values * 0.0001 to 0.05.

Subsequent experiments were conducted to test the hypothesis that phagocytic cells may have enhanced the process from phagocytosis to digestion through alteration of the phenotype by responding rapidly to an enormous bacterial load. Intraperitoneal cells were harvested from the CLP model and used as material for studies ranging from phagocytosis to lysosomes. Although the lysosome contains hydrolases that digest pathogens and macromolecules, it is necessary to expand the volume of the phagolysosome and maintain its lumen at approximately 4.6 pH, the optimum for these hydrolases, to process large amounts of pathogens. First, Tfeb mRNA expression was examined, which was markedly decreased by CLP alone and significantly increased by treatment with ZLN005 but was still lower than that in the sham group (**Figure 5A**). Lysosomal mass was increased by CLP alone and further enhanced by treatment with ZLN005 (**Figure 5A**, **Supplementary Figure 6A**). Intraperitoneal cells were incubated with fluorescent dextran, and the fluorescence intensity was evaluated as the degree of phagocytosis. Phagocytosis was significantly elevated in the CLP group, and ZLN005 showed further elevation, although no significant difference was recognized (**Figure 5B**, **Supplementary Figure 6B**). Next, we examined the final stage of bacterial killing and the intraluminal alterations of the lysosome. Lysosome pH is one of the most fundamental requirements for lysosome function, and the enhancement of acidity was measured as an increase in fluorescence intensity using a pHrod. Lysosomal acidity was enhanced in the CLP group and was further significantly enhanced by treatment with ZLN005 (**Figure 5C**, **Supplementary Figure 6C**). However, the expression of intracellular ROS, as measured by CellRox, was not significantly elevated during this period (**Figure 5D**, **Supplementary Figure 6D**). The acidic shift of the lysosome pH was linked to the promotion of the function of hydrolases, as shown by the use of DQ-BSA, in which the fluorescent dye is released from quenching inhibition and excites fluorescence when proteolysis is enhanced (**Supplementary Figure 7E**). Although a certain degree of proteolysis occurs in unstimulated cells by the addition of DQ-BSA, the degree of proteolysis is enhanced by LPS exposure, and is further enhanced by the administration of ZLN005, as observed by fluorescence microscopy (**Figure 5E**, **Supplementary Figure 6F**), and quantification by FACS showed that ZLN005 promoted proteolysis with significant differences (**Figure 5E**, **Supplementary Figure 6G**). These results suggest that in the present model, the oxidative burst is not significantly involved in pathogen eradication after 1 day of infection, but rather the hydrolytic processing is considered to be a major contributor.

The mRNAs of most lysosomal proteins that possess the CLEAR motif in the promoter, which is a target of TFEB (45), were elevated compared with those in the CLP group. In particular, all mRNAs of hydrolases, such as Ctsd, and membrane proteins, such as Atp6v1A, Atp6v0d1, and Mcoln1, were significantly elevated (**Figure 5F**). With respect to autophagy, Becn1 and Gabarap were only slightly elevated, and Rab7 was not changed by the administration of ZLN005, although a significant increase was recognized on Sqstm/p62 (**Figure 5G**). The analyses of these transcripts suggest that ZLN005 could be more involved in phagolysosome acidification than in xenophagy. Bacteria killing in phagocytes can be achieved by fusion of phagosomes with lysosomes or by xenophagy, in which the host triggers autophagy when the phagosomes are damaged by the escape behavior of the bacterium. We investigated whether the increase in lysosomal acidity and activation of hydrolase activity by ZLN005 could lead to the activation of xenophagy using the following methods. In the LPS model of THP-1, we examined autophagy flux in the presence of bafilomycin A1, an ATPase inhibitor, and chroloquine, a fusion inhibitor of autophagosomes and lysosomes, using LC3 conversion as an indicator. The autophagy flux was measured after 6 hours of exposure to LPS followed by 2 or 6 hours of treatment with the autophagy inhibitor (**Supplementary Figure 7A**). Neither LC3-II nor LC3-II/I ratios were increased by treatment with ZLN005 (**Figure 5H**). The effects of the two inhibitors were common in this experimental system, with LC3-II and LC3-II/I ratios increasing with significant differences, while ZLN005 had no significant effect on the increase of the two inhibitors to LC3-II and LC3-II/I ratios. These results indicate that the effect of ZLN005 on macroautophagy is not significant.

### Regulation of TFEB

**Figure 6:**
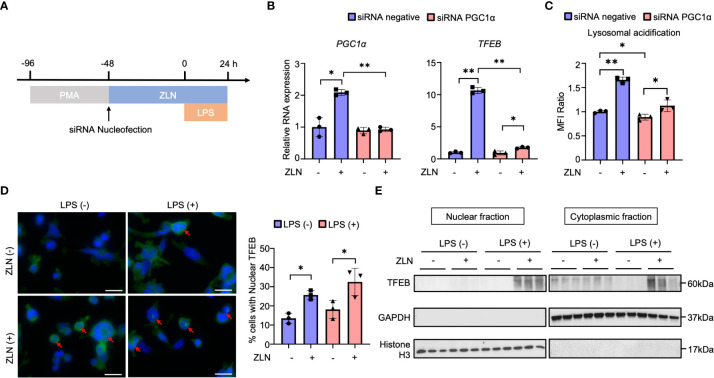
**(A)** Protocol of the PGC1α gene knockdown by siRNA nucleofection in THP-1 cells. **(B)** The mRNA expression of *PGC1α* and *TFEB* at 72 hours post-siRNA nucleofection (n = 3). **(C)** The assessment of lysosomal acidification in THP-1 cells at 72 hours post-siRNA nucleofection. **(D)** TFEB and DAPI stained confocal microscope images of THP-1 cells in the inflammation model at 24 hours post-LPS administration. Arrows indicate TFEB transitioned to the nucleus. All bars in the images are 20 μm. The graph shows the ratio of the cells with nuclear TFEB (n = 3). **(E)** Western blot analysis for TFEB in the nuclear and cytoplasmic fraction of THP-1 cells in the inflammation model at 24 hours post-LPS administration (n = 3). P values ** < 0.0001, P values * 0.0001 to 0.05.

We investigated how the action of ZLN005 on lysosomes is related to its action as a PGC1α activator and what molecular mechanism is responsible for the lysosomal alteration. PGC1α KD did not significantly change the mRNA level of Tfeb in THP-1 with LPS stimulation alone. When ZLN005 was added to LPS stimulation, Tfeb mRNA levels were markedly increased, but PGC1α KD caused an increase in Tfeb mRNA levels, but the rate of increase was greatly reduced (**Figures 6A, B**). These results suggest that PGC1α plays a role in the regulation of TFEB at the transcriptional level upon treatment with ZLN005. Lysosomal acidification was not altered by PGC1α KD when stimulated with LPS alone, and was significantly elevated when ZLN005 was administered, although the increase in acidity was slightly reduced (**Figure 6C**). This suggests that ZLN005 may have a mechanism of action other than transcriptional regulation of Tfeb.

Alternatively, Tfeb is always present in the cytoplasm, and its nuclear translocation is regulated by phosphorylation, where it acts as a transcription factor. We considered if there might be intervention of ZLN005 in this pathway and performed fluorescent immunostaining of TFEB in THP-1 by LPS exposure and ZNL005 administration (**Figure 6D**, **Supplementary Figure 7B**). Quantification showed that nuclear migration proceeds in the absence of LPS exposure by ZLN005, but is further enhanced by LPS exposure with ZLN005 (**Figure 6D**). To further confirm the nuclear migration of TFEB, cells were collected and divided into nuclear and cytoplasmic fractions and Western Blotting was performed to examine the presence of TFEB; in the presence of LPS exposure, nuclear migration of ZLN005-induced TFEB was quite pronounced (**Figure 6E**).

**Figure 7:**
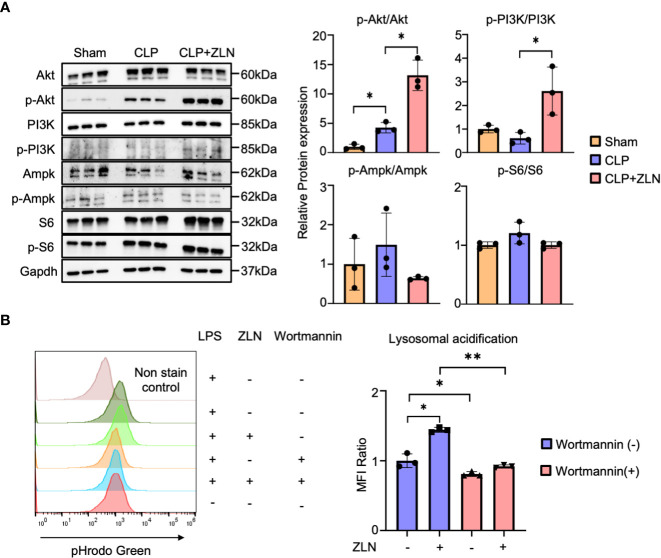
**(A)** Western blotting analysis of the molecular pathways of peritoneal cavity cells at 24 hours post-CLP. The graph shows the expression ratio of each protein corrected by Gapdh protein expression level (n = 3). **(B)** The assessment of lysosomal acidification in THP-1 cells at 5 days post-Wortmannin administration. The statistical significance of differences among two groups, with or without ZLN005 in Wortmannin administration, were evaluated using Kolmogorov-Smirnov test. P values ** < 0.0001, P values * 0.0001 to 0.05.

Finally, we examined the molecular pathway of action in ZLN005 and found that among the various factors that promote V-ATPase V0-V1 assembly, the PI3K/AKT axis is the pathway most closely involved in phagocytosis. The involvement of mTORC1, which is downstream of this pathway, and AMPK, which is closely linked to the regulation of both PGC1α and TFEB, was also investigated using WB, demonstrating that PI3K and AKT were markedly phosphorylated and activated by ZLN005 treatment (**Figure 7A**). However, no activation of S6 downstream of mTORC1 was observed, and the activity of AMPK was hardly changed by ZLN005 administration (**Figure 7A**). Both mTORC1 and AMPK are deeply involved in autophagy regulation, and ZLN005 did not activate either of them. Since the PI3K-AKT pathway is greatly activated by ZLN005, which may play a major role in the phosphorylation of TFEB, we tested whether the PI3K inhibitor Wortmannin could cancel the change in lysosomal acidification. ZLN005 did cause acidification of lysosomes in the absence of Wortmannin, but did not cause acidification in the presence of Wortmannin (**Figure 7B**). These results suggest that ZLN005 causes lysosomal acidification through PI3K, which could promotes V0-V1 assembly.

### Mode of action of ZLN005

**Figure 8:**
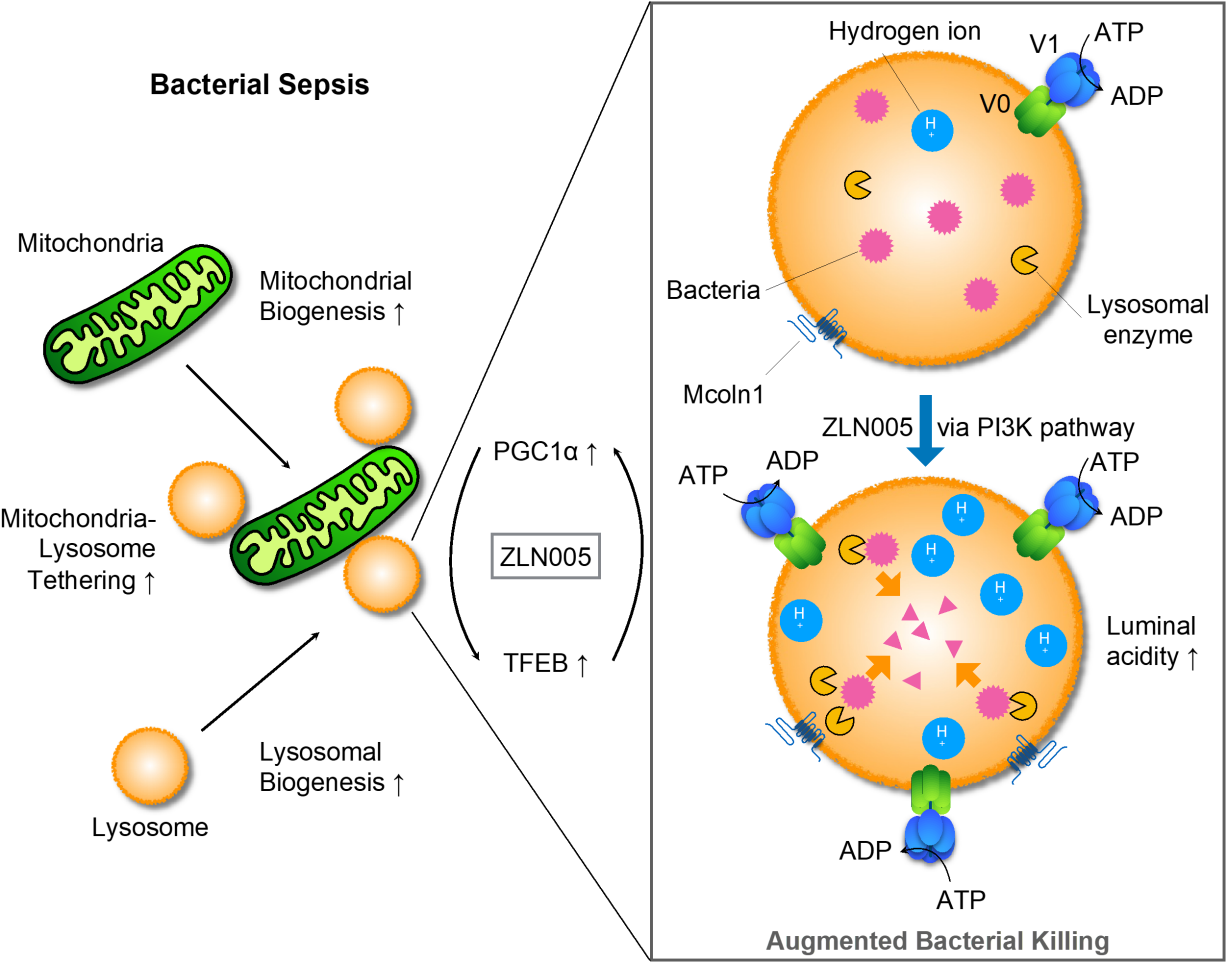
Graphic schema for the mode of action of ZLN005 to polymicrobial sepsis.

For mitochondria, ZLN005 causes mitochondrial biogenesis, which is transcripitionally regulated *via* PGC1α. On the other hand, ZLN005 makes lysosomes promptly increased luminal acidity with enhanced lysosomal biogenesis *via* TFEB nuclear translocation. Hydrolases increased by ZLN005 could effectively exerted bacteriocidal effects and supported bacterial clearance (**Figure 8**).

## Discussion

In this study, we confirmed that intraperitoneal administration of ZLN005, which has been reported as a PGC1α agonist, is therapeutic for polybacterial sepsis and showed that the molecular mechanism of the effect is the enhancement of lysosomal acidity and biogenesis through TFEB. The upstream signal for TFEB activation could be PI3K. ZLN005 has a dual action on two intracellular organelles, mitochondria and lysosomes, and their mechanism of action could be coordinated. For bacterial clearance in the early phase of sepsis, ZLN005 has a mechanism of action that contributes to the improvement of the disease state.

Among interventions related to mitochondrial function in the development of therapies for sepsis, the pathway involving PGC1α, the master regulator of mitochondrial biogenesis, is one of the most intensively studied (14, 46). PGC1α is positively regulated both at the transcriptional level by AMPK and at the posttranslational level through phosphorylation (47). AICAR, an AMPK agonist, acts on PGC1α through activation of SIRT1 and reduces cardiac dysfunction in CLP models (48), while Compound C, an AMPK antagonist, enhances tissue damage associated with sepsis (49). Metofolmin has been shown to stimulate AMPK and increase PGC 1α (50) and has been shown to be organ protective in CLP models (51). A clinical meta-analysis showed that patients taking metofol minutes before sepsis had a lower mortality rate due to sepsis (52). PPARγ, which is downstream of PGC1α, is a nuclear transcription factor. The PPARγ agonists pioglitazone (53), ciglitazone (54) and troglitazone (55) showed reduced inflammation and improved survival in the CLP model. Fenofibrate, a PPARα agonist, also enhanced bacterial clearance in sepsis caused by Salmonella typhimurium (56). ZLN005 showed anti-inflammatory activity against Pseudomonas aeruginosa infection by suppressing inflammasomes through mitochondrial Sirt3 (57). Regarding the mode of action, the study that reported ZLN005 showed slow kinetics with a 2.5-fold increase in PGC1α mRNA expression at 48 hours (58). ZLN005 has been reported to be useful in suppressing kidney fibrosis (59) and reducing ischemia-reflux injury (60) through regulation of mitochondrial function. However, while the former is considered reasonable based on the gradual changes in transcriptional regulation and the time axis, the latter is a condition that causes extremely rapid changes from seconds to minutes. Therefore, the mode of action of ZLN005 was suspected to involve interventions in posttranslational regulation or protein−protein interactions. In this study, the induction of Tfeb mRNA by ZLN005 under LPS stimulation was suppressed by PGC1α KD, indicating that Tfeb mRNA is regulated by PGC1α. On the other hand, the enhancement of TFEB migration to the nucleus by ZLN005 treatment was observed by immunofluorescence staining and western blotting analyses in the nuclear and cytoplasmic fractions, suggesting that ZLN005 acts directly on the nuclear migration of TFEB. The action of ZLN005 on lysosomes *via* TFEB, in addition to the reciprocal enhancement of PGC1α at the transcriptional level, may be a mechanism to promote nuclear translocation of TFEB that is sustained for a period of time from very early pathological changes.

V-ATPase, the molecule responsible for lysosomal acidification (61), has been shown to be regulated by V0-V1 reversible disassembly, but its role in pathogenesis has been largely focused on cancer (62). It is significant impact that this study sheds light on the role of V-ATPase in sepsis. The disassembly is caused by sugar depletion (63). Whereas, other factors that promote V0-V1 reassembly include amino acid depletion (64), ERK (65), EGF (66), and PI3K/AKT (67). PI3K is adjacent to the intracellular domain of receptors for phagocytosis (26). This signal is also involved in the regulation of intracellular vesicle lumen pH through phosphorylation of phosphatidylinositol (32). How PI3K regulates V0-V1 assembly has not been elucidated, but two molecules have recently been reported to be involved in its regulation.Rabconnectin-3, which play a role of chaperon to take free V1 to lysosomal membrane engaged V0, is involved in the reassembly (63). Another is TRiC, which functions to hold the V1 complex in cytosol (68). In sepsis, the inability of the lysosomal system to adequately respond to excessive bacterial load could be a factor in early mortality, and the dysfunction of V0-V1 ATPase assembly might be the molecular mechanism responsible for this inability. In this study, ZLN005 promoted bacterial killing to enhance survival with strengthening lysosomal acidity, suggesting that one of the molecular bases of innate immunity to sepsis is the V0-V1 assembly.

Considering that the effect of ZLN005 on macroautophagy and mitophagy is limited in the THP-1 model using LPS, and that ZLN005 also has only a slight enhancing effect on macroautophagy gene expression in the intraperitoneal cells of the CLP model, ZLN005 has only a limited effect on autophagy in the early stages of infection. The pathway by which ZLN005 improved survival in the CLP model appears to be by promoting bacteria killing in the phagolysosomes. Regarding what directly executes the process in the final stages of bacterial killing, lysosome acidity, luminal hydrolases, and ROS are considered (69). A fungus ensures intracellular survival by alkalinizing lysosomal acidity (70), and it has been shown that attenuation of lysosome acidity reduces bactericidal activity against *E. coli* (71) and *Staphylococcus aureus* (72). However, many bacteria survive well at pH 2-3, which is lower than the pH of the lysosome (73), suggesting that it is unlikely that acidity itself is bactericidal. Rather, it is thought that hydrolases, which set the acidic environment of the lysosome to the optimum pH, play a major role and contribute significantly to bactericidal activity (74). Oxygen radicals and peroxynitrites in the lumen associated with the oxygen burst after infection are also thought to play a major role in bactericidal activity (61). The lack of a significant effect of ZLN005 on cellular ROS and the significant increase and activation of hydrolases suggest that the bacterial clearance could be attributed to hydrolases, rather than cellular ROS.

ZLN005 strongly induced TFEB, a master gene that stimulates the production of membrane proteins and hydrolytic enzymes in the lysosome, which in turn act as an increase in lysosomal mass. TFEB is a transcriptional regulator with a positive feedback loop that rapidly responds to environmental changes mainly by posttranslational modification and transcriptionally enhances PGC1α (75). The stability and translocation of TFEB from the cytoplasm to the nucleus is regulated by phosphorylation by various kinases and dephosphorylation by phosphatases, and it translocates to the nucleus to promote the expression of its own genes and genes involved in autophagy and lysosome biogenesis. Many signal regulators negatively regulate TFEB, including mTORC1 (76), but calcineurin (77) positively regulates TFEB. PIKFYVE on the lysosome is a kinase that receives signals from AKT (78), which substrates PI3P to generate PI(3,5)P2 (79). It has been reported that PI(3,5)P2 activates TRPML1, which is encoded by MCOLN1, and TRPML1 releases Ca^2+^ from lysosomes into the cytoplasm. Its increased concentration activates calcineurin, resulting in the dephosphorylation of TFEB to act as a transcription factor (80). The involvement of PI3K in the mechanism of action of ZLN005 was indirectly demonstrated by enhanced phosphorylation of PI3K and AKT in the CLP model. In addition, lysosomal acidificationby ZLN005 was inhibited by the PI3K inhibitor wortmannin, providing direct evidence that ZLN005 acts through PI3K. These factors, including PIKFYVE, TRPML1, and calcineurin, may be involved and contribute to the activation of TFEB in ZLN005. Signals from PI3K are transduced *via* AKT to mTORC1, which leads cells to anabolism, including proliferation, through phosphorylation of various factors (81). However, mTor signals suppress all autophagy processes, not only initiation and nucleation but also autophagosome elongation, maturation, and termination (82). Some bacteria have acquired the art of escaping innate immunity by exploiting this mTORC1 suppression of the autophagy process (27). Metabolism in sepsis results in a surge in energy demand, which activates AMPK, and this signal is inhibitory for mTORC1 *via* Rheb through phosphorylation of TSC2 (81). Furthermore, AMPK supports autophagy through phosphorylation of ULK1 independent of mTOR (83). In our experiments, the S6 phosphorylation pathway downstream of mTORC1 was not involved in sepsis pathogenesis, and further activation of PI3K by ZLN005 did not significantly alter the signal downstream of mTORC1. However, the phosphorylation of AMPK, which acts antagonistically with mTORC1, is activated in the sepsis condition but did not change in this study. This is in accordance with the fact that autophagy-related gene expression was only slightly altered in the early phase of sepsis in this study. The fact that signaling from PI3K was involved in lysosomal acidification but had no significant effect on the mTORC1 pathway suggests that the signaling pathway of ZLN005 needs to be further examined in the future.

It was shown in this study that ZLN005 causes an increase in mitochondrial lysosome contact sites and that OXPHOS is enhanced. Physical contact between these two intracellular organelles has long been reported to occur in a mitophagy-like degradative process, but it has recently been recognized that transient contact exists physiologically as a nondegradative process (84). In mitochondrial lysosome contact, approximately 15% of lysosomes are in contact with mitochondria in cell culture, and the contact time is less than 1 minute. The role of this contact site has been shown to be the regulation of each other’s dynamics, and in addition, various metabolites and ions are exchanged (84). If the TFEB response is affected by Ca released from the lysosome to the cytosol, this Ca flows into the matrix *via* VDAC1 in the outer mitochondrial membrane and MCU in the inner membrane at the mitochondrial lysosome contact (85), leading to stimulation of the TCA cycle (86). Morphological changes suggest that there are factors involved in and regulating the alteration of function. Acidification has been reported to affect Drp1 positioning (87), suggesting that luminal acidity may be one of its regulators. The present study shows that LPS-stimulated THP-1 by ZLN005 causes tethering of two organelles from as early as 1 hr to as late as 24 hr. Although the molecular mechanism by which ZLN005 promotes the coordination of the two organelles remains to be elucidated, the enhancement of physical contact might allow the dual function of mitochondria and lysosomes to work more organically.

For sepsis, much effort is focused on controlling the hyperimmune reaction. However, drug development to improve the efficiency of bacterial clearance in the early phase has been stagnant. In this context, ZLN005, which has been shown to improve overall survival by improving the pathophysiology of the early phase of the disease, holds great promise for clinical practice. In addition, ZLN005 will be a great stimulus for drug discovery to intervene in the early phase of sepsis. Furthermore, ZLN005, which has dual action on mitochondria and lysosomes, has potential in the treatment of lysosomal acidification failure-based neurodegenerative diseases, including Alzheimer’s disease, Parkinson’s disease, renal tubular acidosis, diabetes mellitus, Zimmermann-Laband syndrome due to V1B2 mutation, and Cutis laxa type II and wrinkly skin syndrome (34). We believe that diseases that converge on lysosome acidification dysfunction as the molecular basis, albeit in a completely different pathology than sepsis, would be appropriate therapeutic targets for compounds with this action.

## Data availability statement

The raw data supporting the conclusions of this article will be made available by the authors, without undue reservation.

## Ethics statement

The animal study was reviewed and approved by Animal Experiment Ethics Committee of the Kyoto Prefectural University of Medicine (Approval number, M2022-543).

## Author contributions

SG designed the experiments, and analyzed the data. SG and YS wrote the manuscript. YS performed the experiments and analyzed the data with DK. AS, TT, and TO analyzed and discussed the data. SM discussed the clinical relevance. All authors contributed to the article and approved the submitted version.

## Supplementary material

Supplementary Figure 1
**(A)** Schematic illustration of cecal ligation puncture (CLP). **(B)** Survival rate with different needle sizes and numbers of punctures. **(C)** Chemical structure of ZLN005. **(D)** Protein and mRNA expression of PGC1α in THP-1 cells at 0, 12, 24, 36 and 48 hours after ZLN005 administration (n = 3).

Supplementary Figure 2
**(A)** Representative examples of hematoxylin-eosin-stained mouse hearts at 24 hours post-CLP. **(B, C)** Representative images of colony-forming units (CFU) and measurement of bacterial colony number in peritoneal lavages at 2 hours **(B)** and 24 hours **(C)** post-CLP. **(D)** C57BL/6 Mice were injected intraperitoneally with Vehicle or ZLN005 immediately after CLP and peritoneal lavages was collected 2 or 24 hours later. The peritoneal lavages collected 2 hours post-CLP was undiluted, while the fluid collected 24 hours post-CLP was diluted 1000-fold and incubated in LB agar for 24 hours.

Supplementary Figure 3
**(A)** Protocol of the inflammation model in THP-1 cells. The experimental protocol for this schema was used to derive the results in . **(B, C)** MFI ratio and histogram of mtMass **(B)** and mtMP **(C)** of THP-1 cells in the inflammation model at 24 hours post-LPS administration (n = 3). **(D)** Histogram of mtROS levels in THP-1 cells in the inflammation model at 24 hours post-LPS administration. **(E)** Protocol of the assessment of cellular ROS in THP-1 cells. **(F)** MFI ratio of cellular ROS in THP-1 cells in the inflammation model at 0, 2, 4, 8, 16 and 24 hours post-LPS administration.

Supplementary Figure 4
**(A)** The construction of pMXs-MTS-mKeima Red. **(B)** The process of purifying mKeima Red-expressing THP-1 cells. **(C)** Protocol of the mitophagy detection assay in mKeima Red-expressing THP-1 cells. The experimental protocol for this schema was used to derive the results in . **(D)** FACS analysis of acidic mKeima Red-positive cells at 0, 4, 8 and 24 hours post-LPS administration. Acidic mKeima Red was set at 561-nm (<pH 6) lasers with 615/20-nm emission filters.

Supplementary Figure 5
**(A)** Workflow of mitochondrial network analysis**. (B)** Fluorescence microscopy images of mitochondria (green), lysosomes (red) and their contact sites (yellow). All bars in the images are 50 μm. **(C)** Flowchart for analyzing the proximity of mitochondria and lysosomes.

Supplementary Figure 6
**(A-D)**, Histogram of lysosomal mass **(A)**, phagocytosis **(B)**, lysosomal acidification **(C)**, and cellular ROS **(D)** at 24 hours post-CLP. **(E)** Schematic illustration shows how DQ Green BSA fluoresces. **(F)** Degradation of lysosomally preloaded DQ BSA in peritoneal cavity cells at 24 hours post-CLP. Representative images are shown 3 hours after incubation with DQ Green BSA. All bars in the images are 100 μm. **(G)** Histograms of lysosomal proteolysis estimated by FACS.

Supplementary Figure 7
**(A)** Protocol of autophagy flux analysis in THP-1. **(B, C)** Western blotting analysis of the autophagy flux in THP-1 cells after 6 hours of exposure to LPS followed by 2 (B) or 6 **(C)** hours of treatment with the autophagy inhibitor. The graph shows the expression ratio of LC3-II protein corrected by α-tubulin protein expression level (n = 3). The graph shows the expression ratio of LC3-II protein corrected by α-tubulin expression level (n = 3). (Con, Control; Baf, Bafilomycin A1; CQ, Chloroquine).

Supplementary Figure 8
**(A)** Histogram of lysosomal acidification in THP-1 cells at 72 hours post-siRNA nucleofection. **(B)** Image analysis process to identify TFEB migrated to the nucleus. **(C)** Protocol of the inflammation model in THP-1 cells. The experimental protocol for this schema was used to derive the results in. **(D)** Protocol of the PI3K inhibition by Wortmannin in THP-1 cells. The experimental protocol for this schema was used to derive the results in.

Supplementary Figure 9 Western blotting membrane for **Figure 5H**.

Supplementary Figure 10 Western blotting membrane for **Figure 6E**.

Supplementary Figure 11 Western blotting membrane for **Figure 7A**.

Supplementary Figure 12 Western blotting membrane for **Figure 7A**.

Supplementary Figure 13 Western blotting membrane for **Supplementary Figure 1D**.

Supplement Movie Post 2 hours appearance of mice treated with CLP either with or without ZLN005 administration.
