# Supplementary figures and images for: ZLN005 improves the survival of polymicrobial sepsis by increasing the bacterial killing via inducing lysosomal acidification and biogenesis in phagocytes
Source: Front Immunol. 2023 Feb 3;14:1089905. doi: 10.3389/fimmu.2023.1089905 (PMC9938763; doi:10.3389/fimmu.2023.1089905)

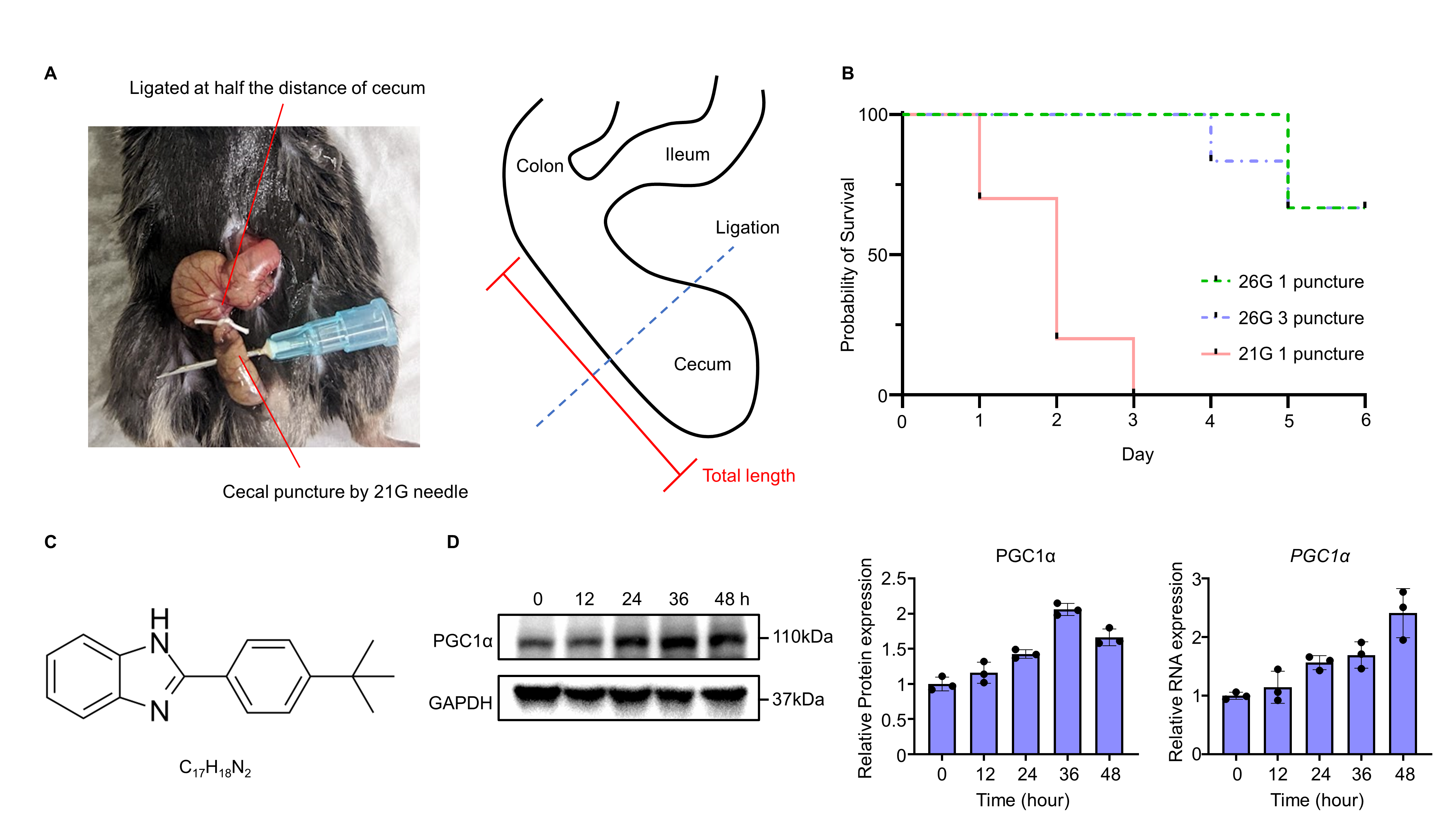

Supplement: Supplementary Figure 1 — (A) Schematic illustration of cecal ligation puncture (CLP). (B) Survival rate with different needle sizes and numbers of punctures. (C) Chemical structure of ZLN005. (D) Protein and mRNA expression of PGC1α in THP-1 cells at 0, 12, 24, 36 and 48 hours after ZLN005 administration (n = 3). [file Image_1.tif]

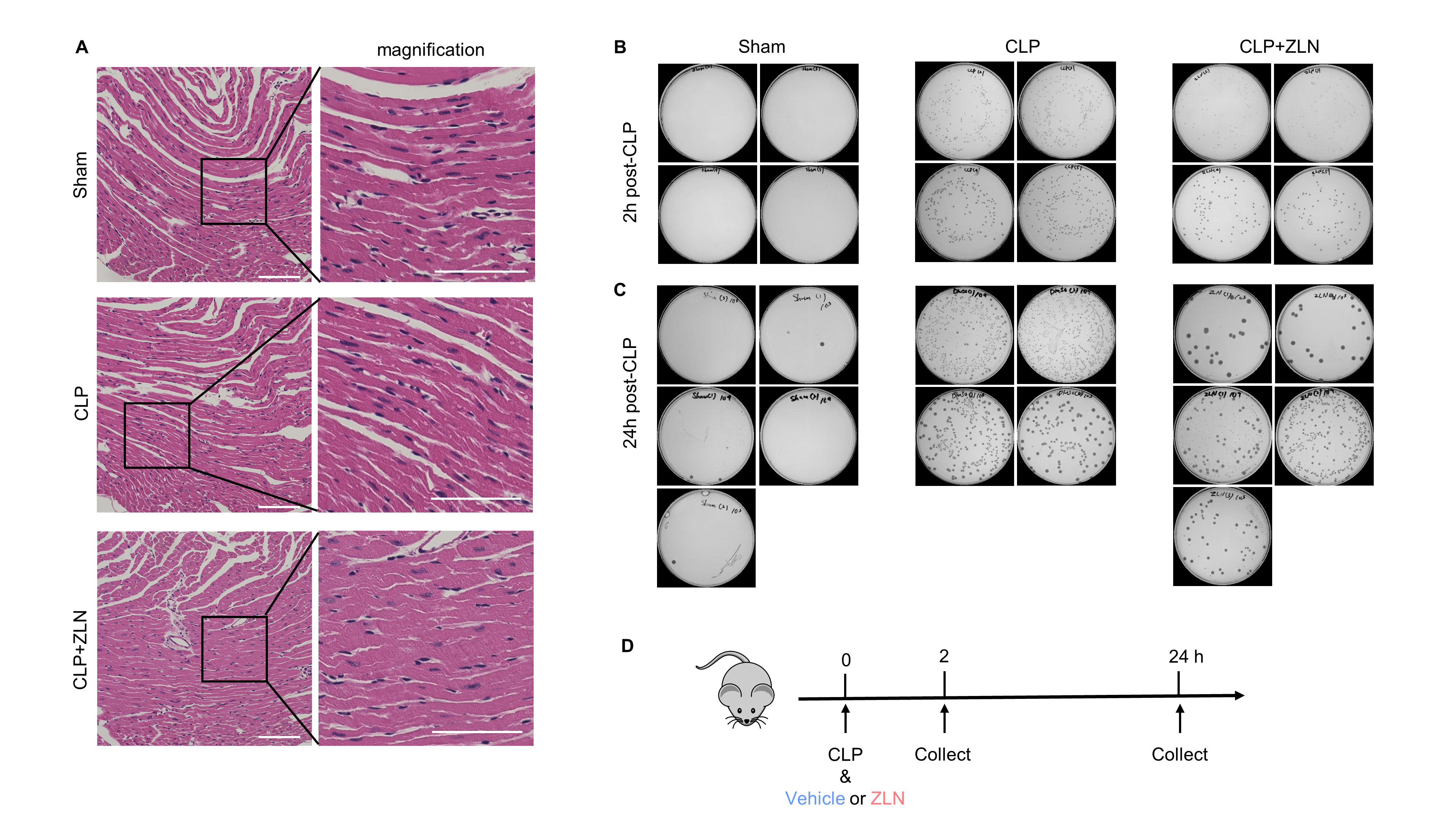

Supplement: Supplementary Figure 2 — (A) Representative examples of hematoxylin-eosin-stained mouse hearts at 24 hours post-CLP. (B, C) Representative images of colony-forming units (CFU) and measurement of bacterial colony number in peritoneal lavages at 2 hours (B) and 24 hours (C) post-CLP. (D) C57BL/6 Mice were injected intraperitoneally with Vehicle or ZLN005 immediately after CLP and peritoneal lavages was collected 2 or 24 hours later. The peritoneal lavages collected 2 hours post-CLP was undiluted, while the fluid collected 24 hours post-CLP was diluted 1000-fold and incubated in LB agar for 24 hours. [file Image_2.tif]

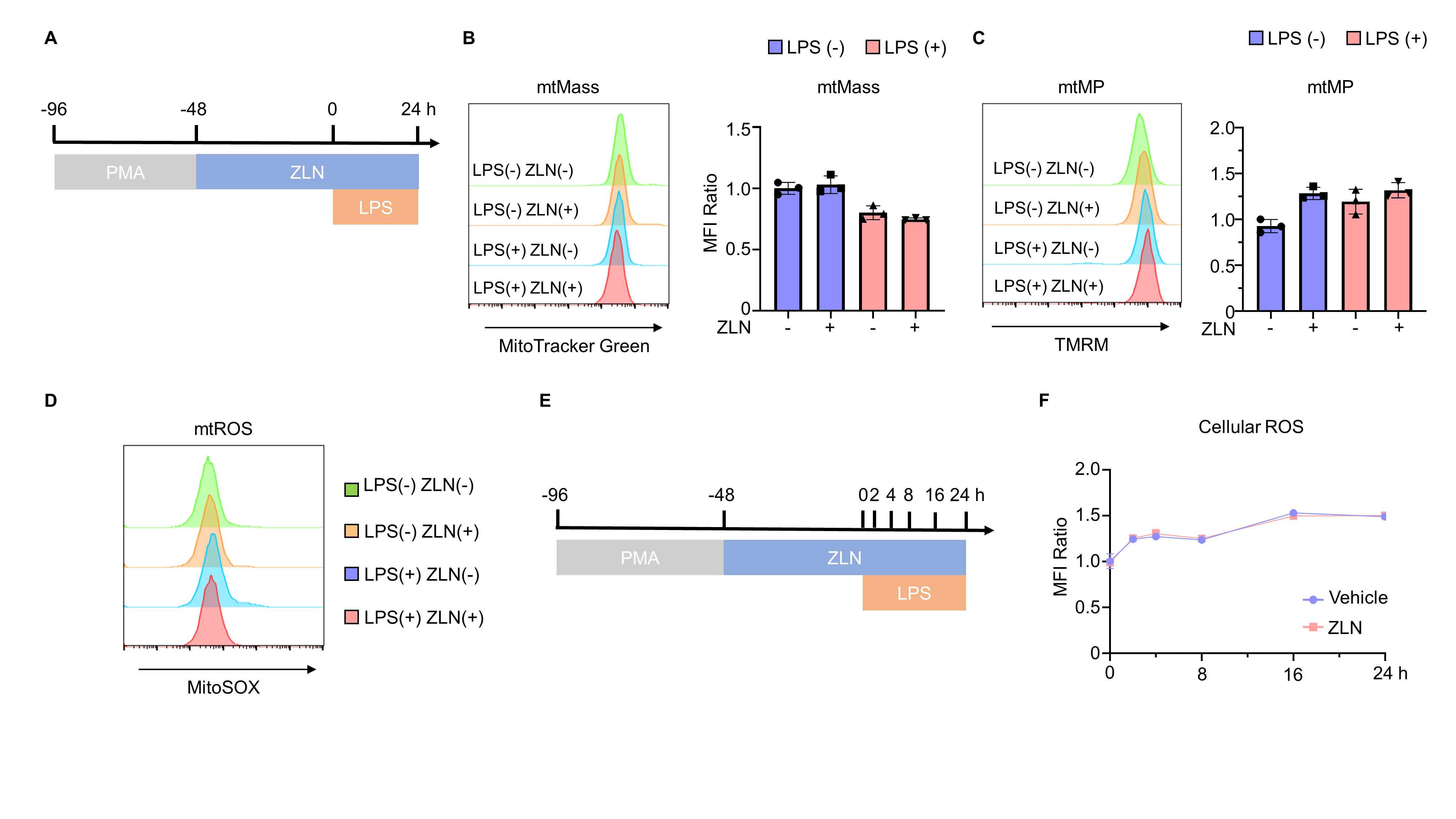

Supplement: Supplementary Figure 3 — (A) Protocol of the inflammation model in THP-1 cells. The experimental protocol for this schema was used to derive the results in . (B, C) MFI ratio and histogram of mtMass (B) and mtMP (C) of THP-1 cells in the inflammation model at 24 hours post-LPS administration (n = 3). (D) Histogram of mtROS levels in THP-1 cells in the inflammation model at 24 hours post-LPS administration. (E) Protocol of the assessment of cellular ROS in THP-1 cells. (F) MFI ratio of cellular ROS in THP-1 cells in the inflammation model at 0, 2, 4, 8, 16 and 24 hours post-LPS administration. [file Image_3.tif]

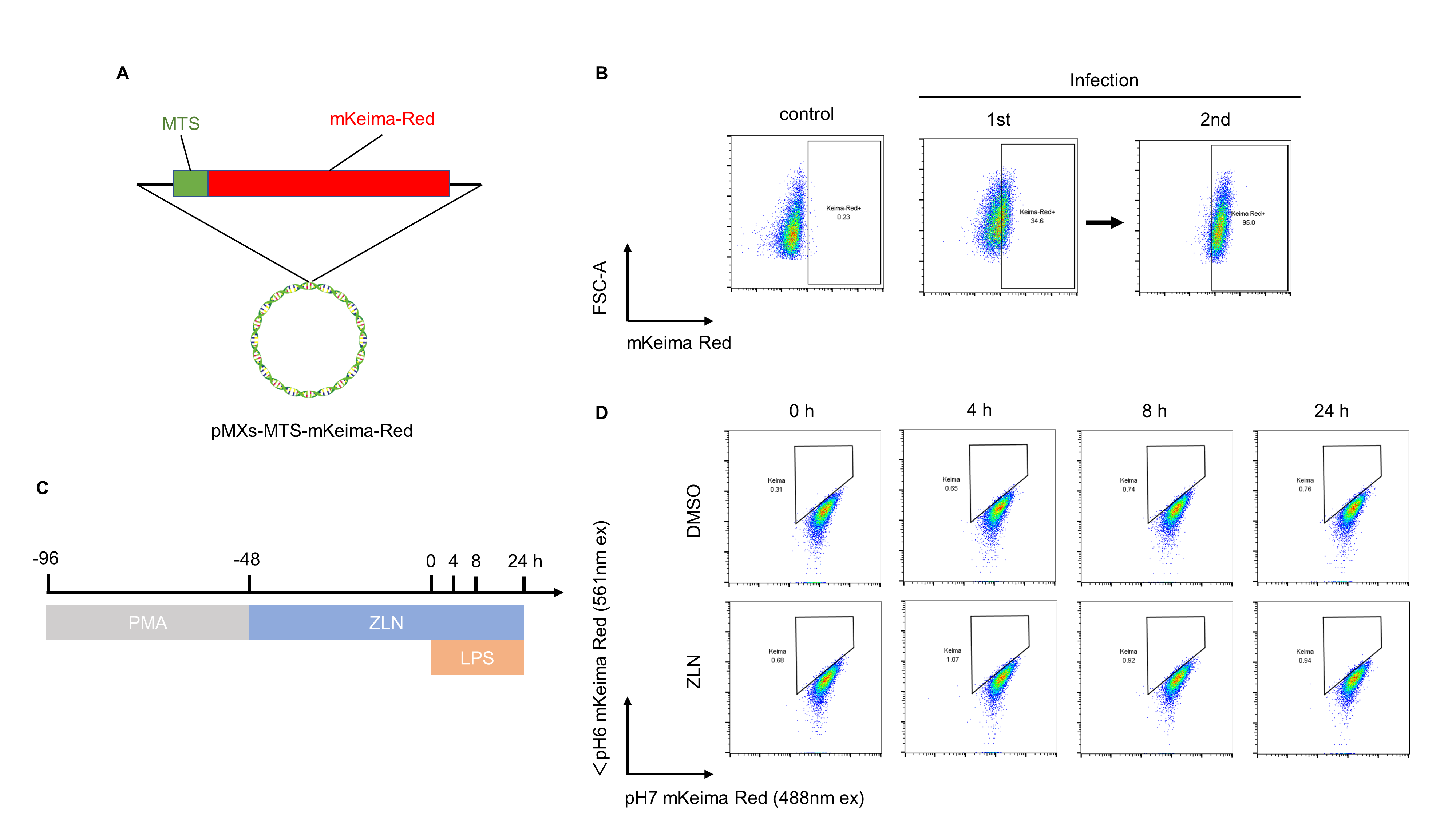

Supplement: Supplementary Figure 4 — (A) The construction of pMXs-MTS-mKeima Red. (B) The process of purifying mKeima Red-expressing THP-1 cells. (C) Protocol of the mitophagy detection assay in mKeima Red-expressing THP-1 cells. The experimental protocol for this schema was used to derive the results in . (D) FACS analysis of acidic mKeima Red-positive cells at 0, 4, 8 and 24 hours post-LPS administration. Acidic mKeima Red was set at 561-nm (<pH 6) lasers with 615/20-nm emission filters. [file Image_4.tif]

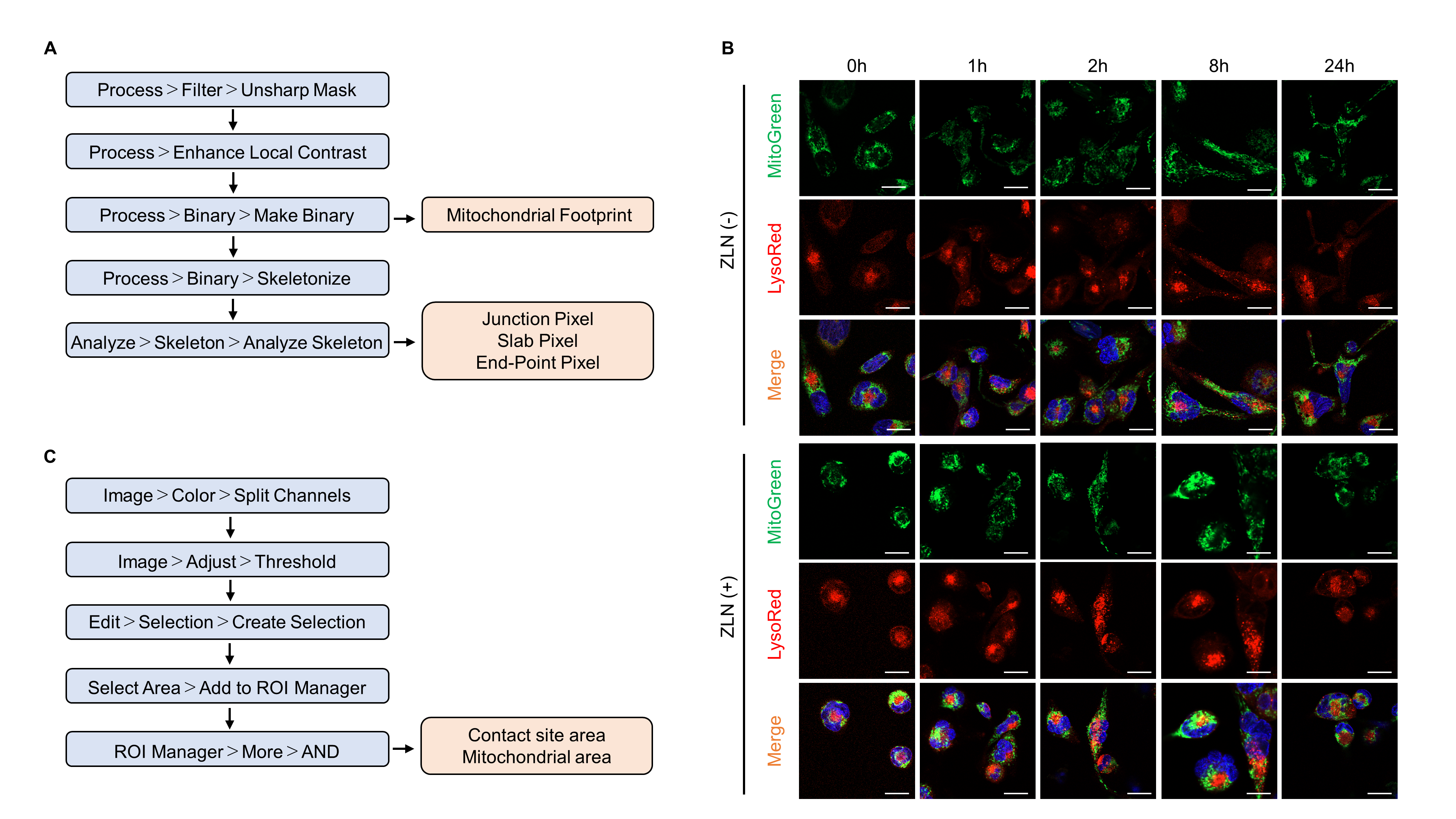

Supplement: Supplementary Figure 5 — (A) Workflow of mitochondrial network analysis. (B) Fluorescence microscopy images of mitochondria (green), lysosomes (red) and their contact sites (yellow). All bars in the images are 50 μm. (C) Flowchart for analyzing the proximity of mitochondria and lysosomes. [file Image_5.tif]

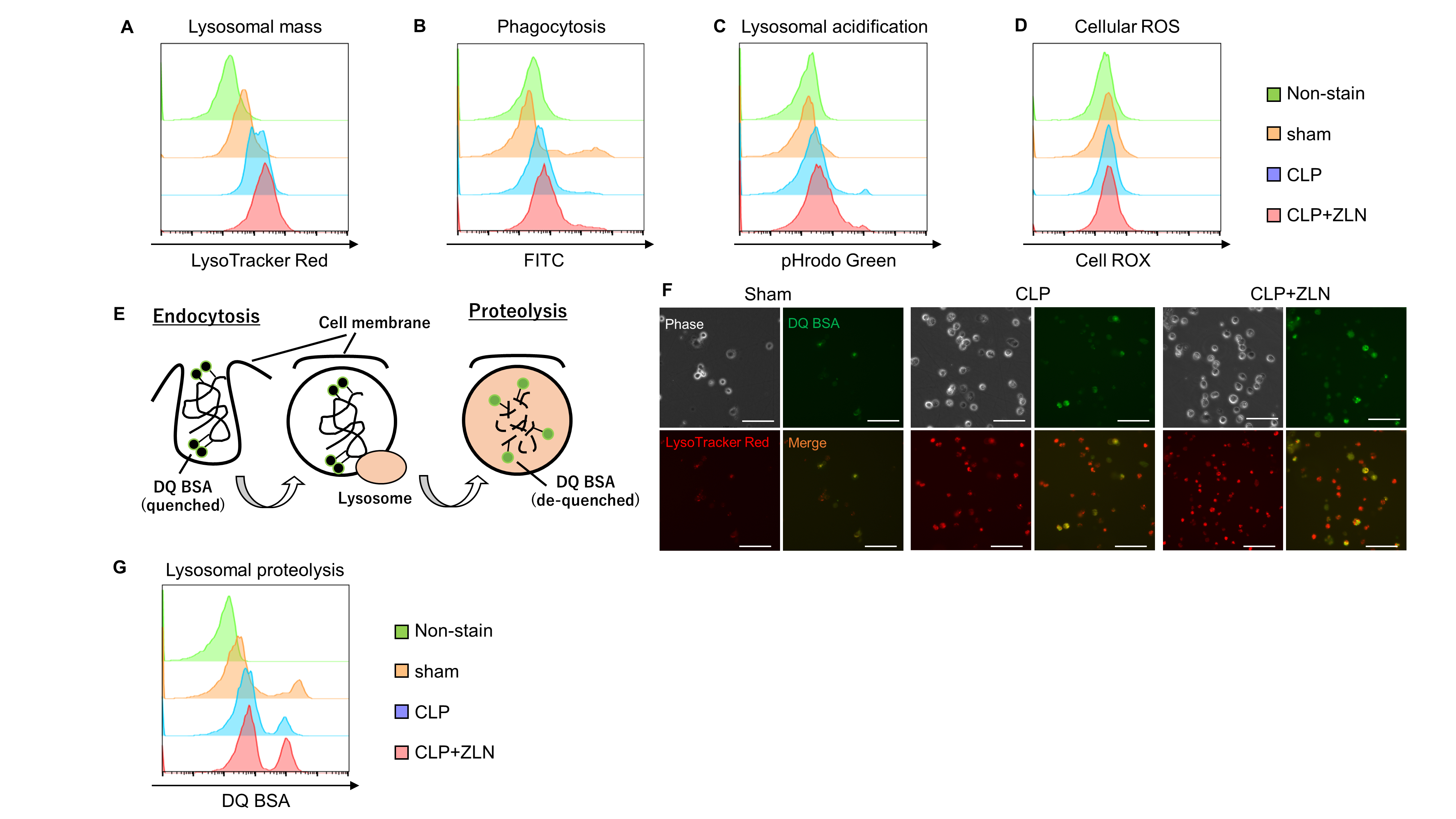

Supplement: Supplementary Figure 6 — (A-D), Histogram of lysosomal mass (A), phagocytosis (B), lysosomal acidification (C), and cellular ROS (D) at 24 hours post-CLP. (E) Schematic illustration shows how DQ Green BSA fluoresces. (F) Degradation of lysosomally preloaded DQ BSA in peritoneal cavity cells at 24 hours post-CLP. Representative images are shown 3 hours after incubation with DQ Green BSA. All bars in the images are 100 μm. (G) Histograms of lysosomal proteolysis estimated by FACS. [file Image_6.tif]

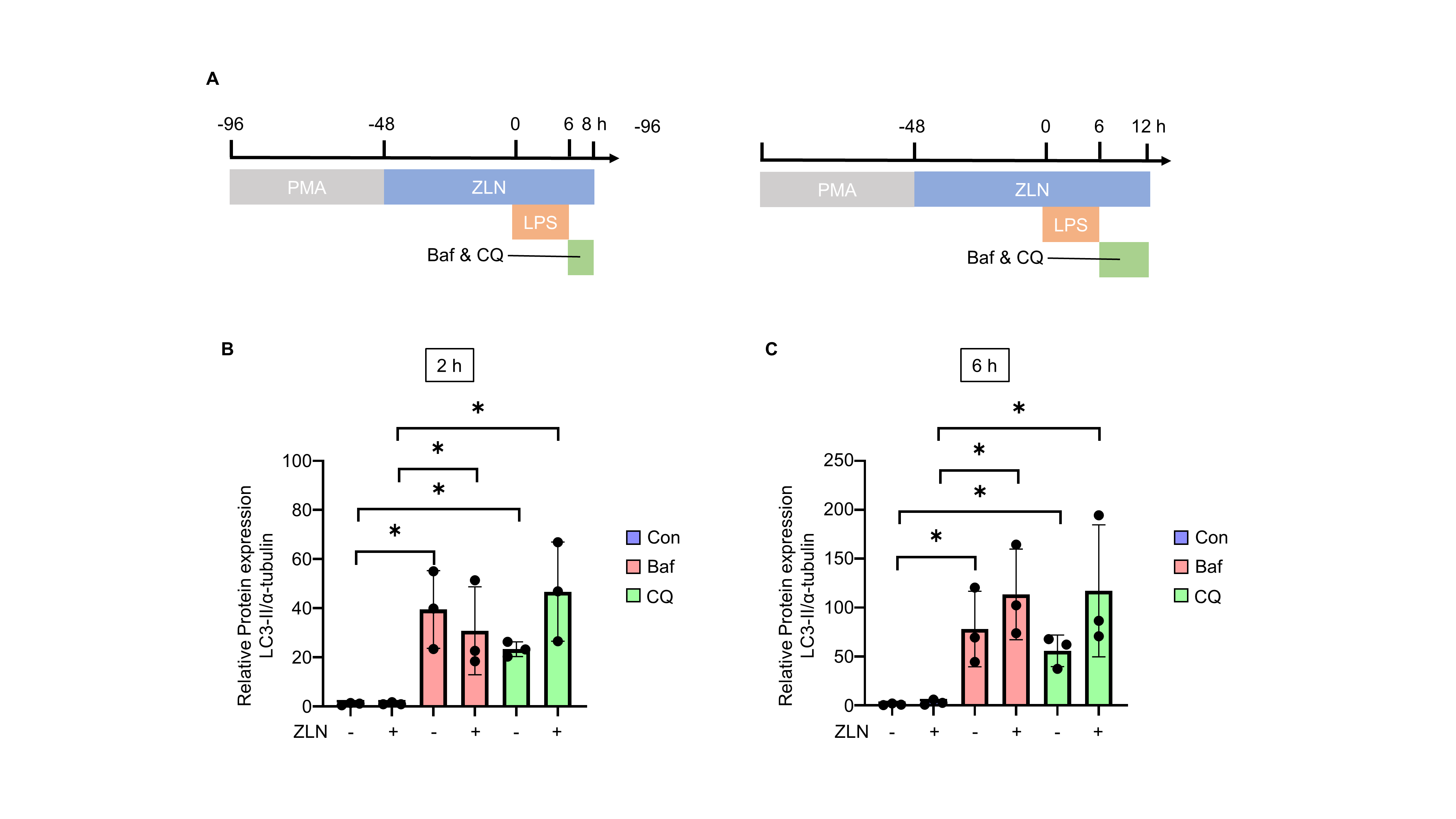

Supplement: Supplementary Figure 7 — (A) Protocol of autophagy flux analysis in THP-1. (B, C) Western blotting analysis of the autophagy flux in THP-1 cells after 6 hours of exposure to LPS followed by 2 (B) or 6 (C) hours of treatment with the autophagy inhibitor. The graph shows the expression ratio of LC3-II protein corrected by α-tubulin protein expression level (n = 3). The graph shows the expression ratio of LC3-II protein corrected by α-tubulin expression level (n = 3). (Con, Control; Baf, Bafilomycin A1; CQ, Chloroquine). [file Image_7.tif]

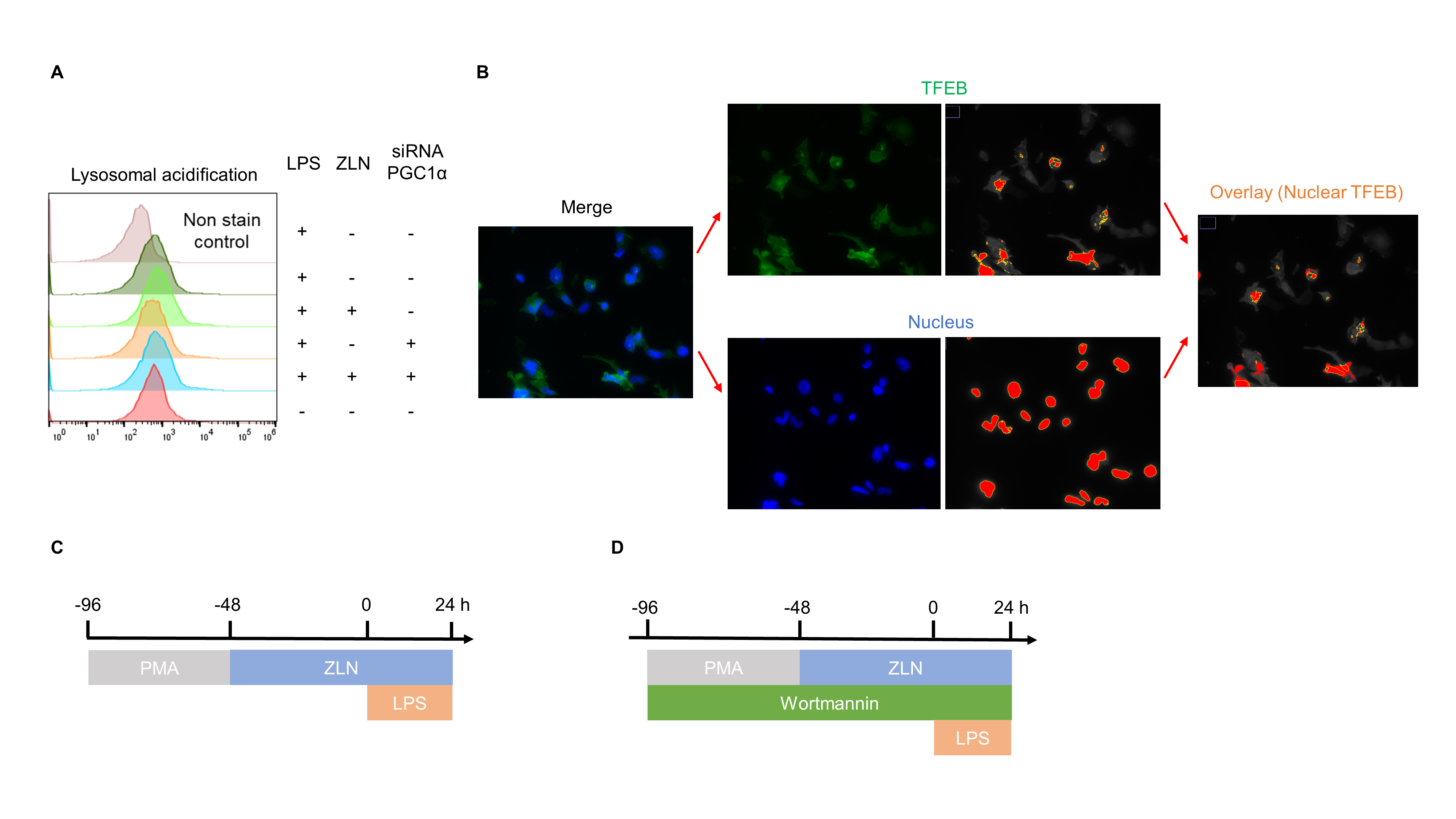

Supplement: Supplementary Figure 8 — (A) Histogram of lysosomal acidification in THP-1 cells at 72 hours post-siRNA nucleofection. (B) Image analysis process to identify TFEB migrated to the nucleus. (C) Protocol of the inflammation model in THP-1 cells. The experimental protocol for this schema was used to derive the results in. (D) Protocol of the PI3K inhibition by Wortmannin in THP-1 cells. The experimental protocol for this schema was used to derive the results in. [file Image_8.tif]

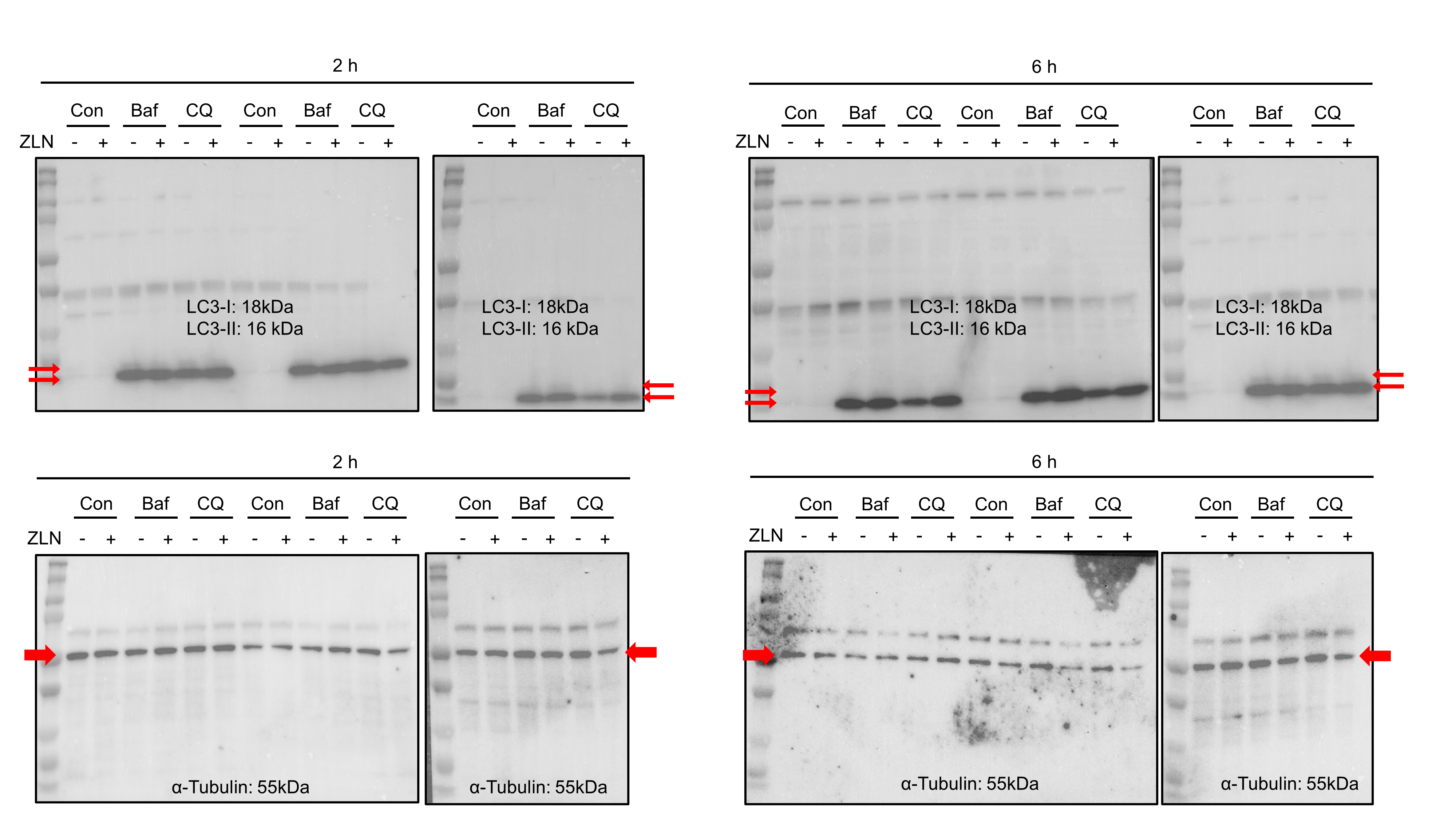

Supplement: Supplementary Figure 9 — Western blotting membrane for Figure 5H . [file Image_9.tif]

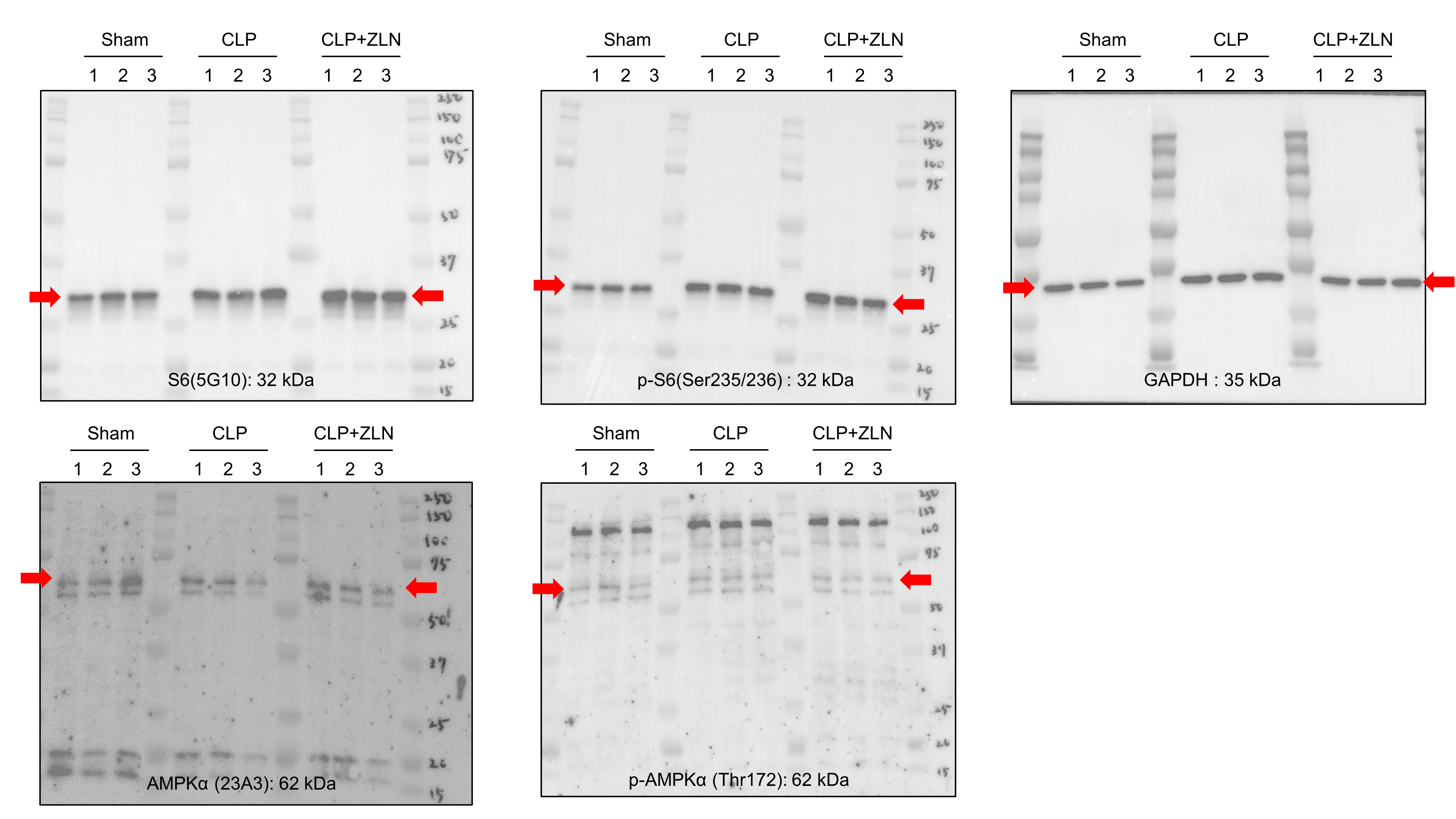

Supplement: Supplementary Figure 10 — Western blotting membrane for Figure 6E . [file Image_10.tif]

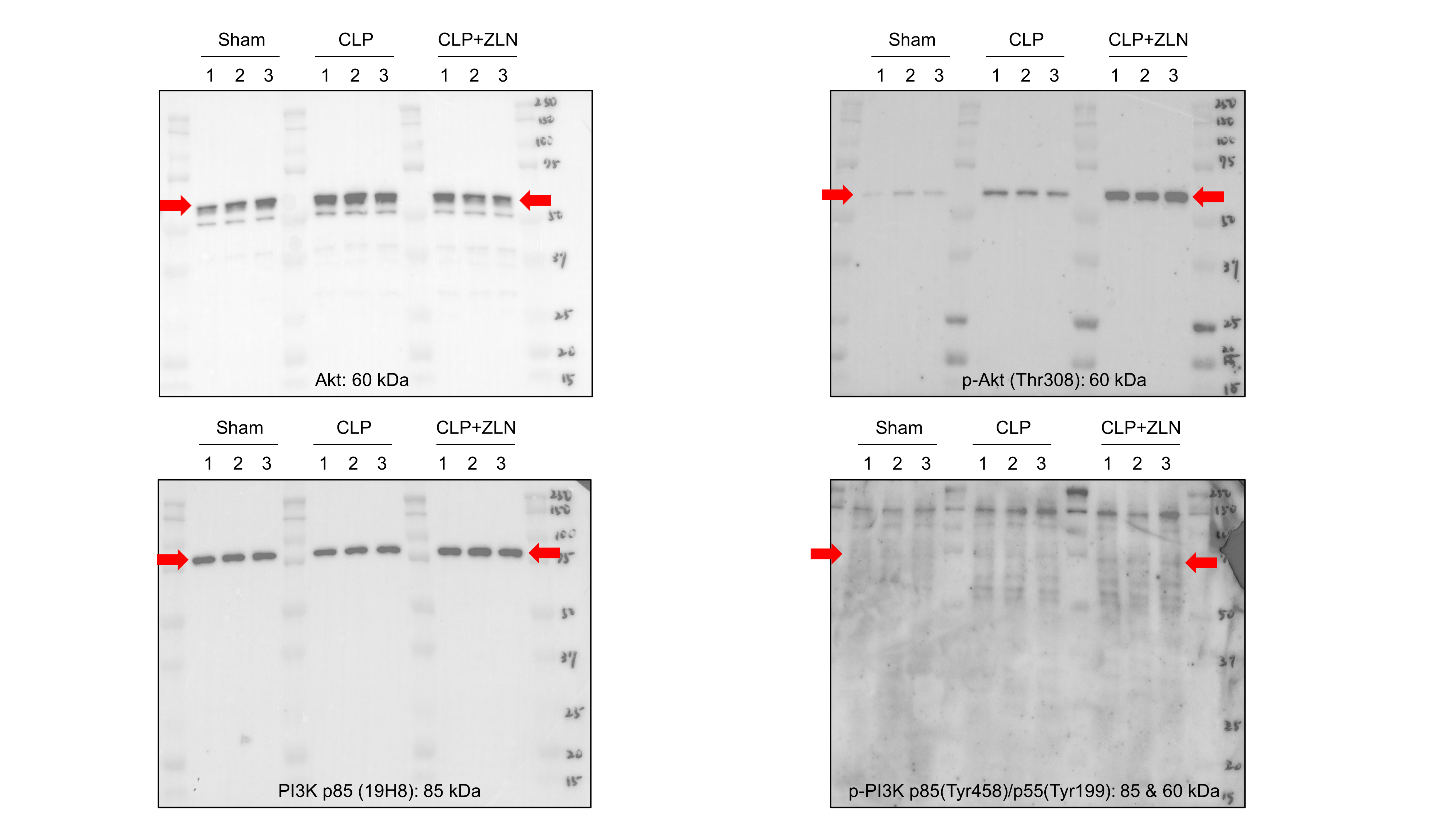

Supplement: Supplementary Figure 11 — Western blotting membrane for Figure 7A . [file Image_11.tif]

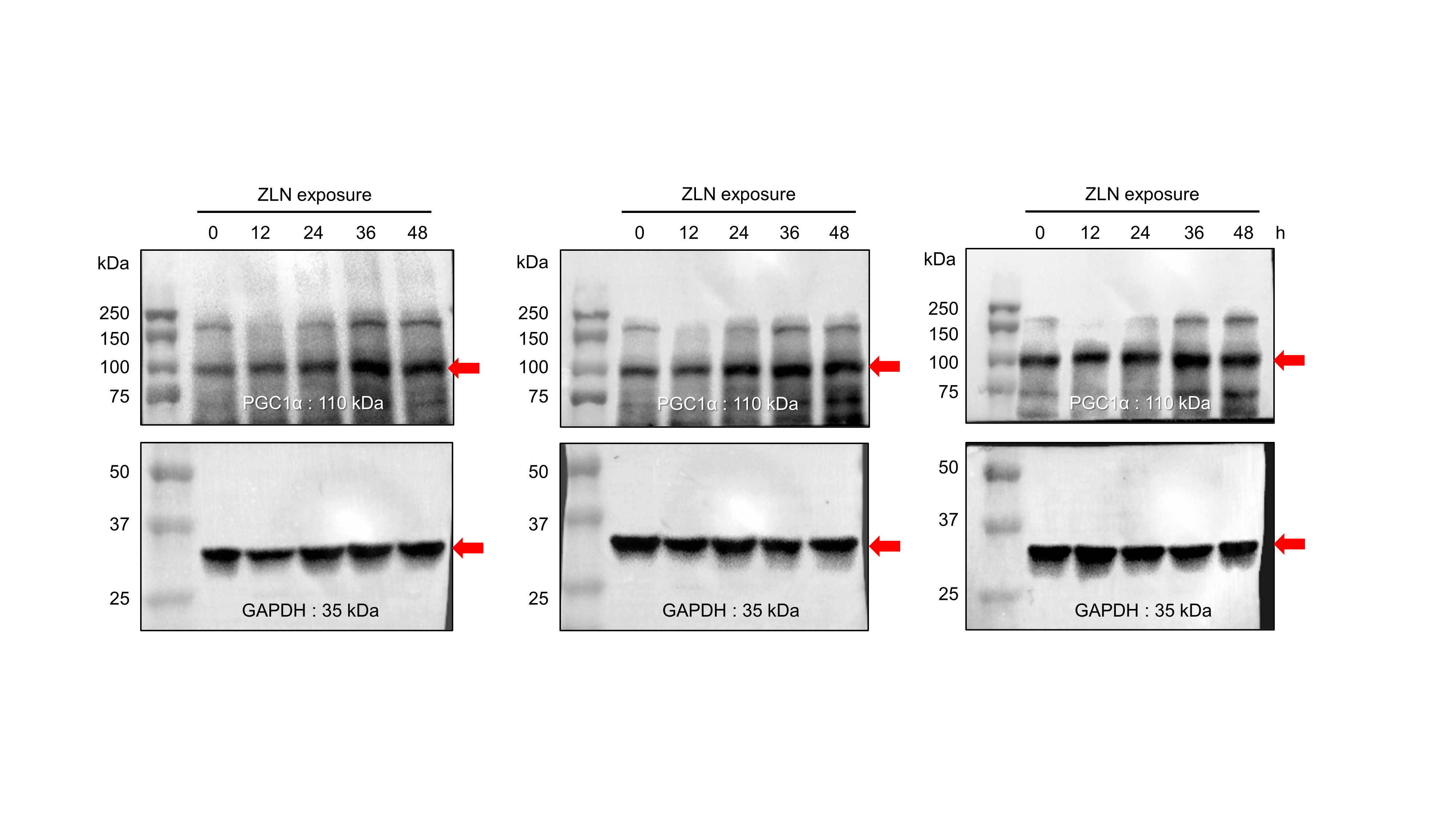

Supplement: Supplementary Figure 13 — Western blotting membrane for Supplementary Figure 1D . [file Image_13.tif]
