# Supplementary material for: ZLN005 improves the survival of polymicrobial sepsis by increasing the bacterial killing via inducing lysosomal acidification and biogenesis in phagocytes
Source: Front Immunol. 2023 Feb 3;14:1089905. doi: 10.3389/fimmu.2023.1089905 (PMC9938763; doi:10.3389/fimmu.2023.1089905)
Supplement: Supplementary file 16 [file Table_1.docx]

**Supplement Table 1 |** Primer sequences, the forward and reverse primer sequences.

| Human | Forward | Reverse |
| --- | --- | --- |
| *PGC1α* | 5′‐TCTGAGTCTGTATGGAGTGACAT‐3′ | 5′‐CCAAGTCGTTCACATCTAGTTCA‐3′ |
| *TFAM* | 5′‐ATGGCGTTTCTCCGAAGCAT‐3′ | 5′‐TCCGCCCTATAAGCATCTTGA‐3′ |
| *GAPDH* | 5’-GCTCAGACACCATGGGGAAGGT-3’ | 5’-GTGGTGCAGGAGGCATTGCTGA-3’ |

| Mouse | Forward | Reverse |
| --- | --- | --- |
| *Tnfα* | 5′‐CAGGCGGTGCCTATGTCTC‐3′ | 5′‐CGATCACCCCGAAGTTCAGTAG‐3′ |
| *Ifnγ* | 5′‐GCCACGGCACAGTCATTGA‐3′ | 5′‐TGCTGATGGCCTGATTGTCTT‐3′ |
| *Il-1β* | 5′‐TTCAGGCAGGCAGTATCACTC‐3′ | 5′‐GAAGGTCCACGGGAAAGACAC‐3′ |
| *Il-6* | 5′‐CTGCAAGAGACTTCCATCCAG‐3′ | 5′‐AGTGGTATAGACAGGTCTGTTGG‐3′ |
| *Pgc1α* | 5′‐TATGGAGTGACATAGAGTGTGCT‐3′ | 5′‐CCACTTCAATCCACCCAGAAAG‐3′ |
| *Tfam* | 5′‐AACACCCAGATGCAAAACTTTCA‐3′ | 5′‐GACTTGGAGTTAGCTGCTCTTT‐3′ |
| *Tfeb* | 5′‐AAGGTTCGGGAGTATCTGTCTG‐3′ | 5′‐GGGTTGGAGCTGATATGTAGCA‐3′ |
| *Hif1α* | 5′‐GATGACGGCGACATGGTTTAC‐3′ | 5′‐CTCACTGGGCCATTTCTGTGT‐3′ |
| *Rab7* | 5′‐AAGCCACAATAGGAGCGGAC‐3′ | 5′‐AGACTGGAACCGTTCTTGACC‐3′ |
| *Gabarap* | 5′‐AAGAGGAGCATCCGTTCGAGA‐3′ | 5′‐GGGGCTTTTTCCACTATCACC‐3′ |
| *Becn1* | 5′‐AGGCGAAACCAGGAGAGAC‐3′ | 5′‐CCTCCCCGATCAGAGTGAA‐3′ |
| *Sqstm1* | 5′‐GAACTCGCTATAAGTGCAGTGT‐3′ | 5′‐AGAGAAGCTATCAGAGAGGTGG‐3′ |
| *Atp6v0d1* | 5′‐TTGAGGGTGCAGGTAGCAATC‐3′ | 5′‐CGATGTTGCGACATTCCTGTTC‐3′ |
| *Atp6V1a* | 5′‐AGCAAAAACCTACGGGTTGGT‐3′ | 5′‐GCTTCCTCTGTTACGTGGGG‐3′ |
| *Gla* | 5′‐TCTGTGAGCTTGCGCTTTGT‐3′ | 5′‐GCAGTCAAGGTTGCACATGAAA‐3′ |
| *Ctsd* | 5′‐CCAGGACACTGTATCGGTTCC‐3′ | 5′‐GCAACAAATACGATTCCAGGCT‐3′ |
| *Mcoln1* | 5′‐GCTGGGTTACTCTGATGGGTC‐3′ | 5′‐CCACCACGGACATAGGCATAC‐3′ |
| *Gapdh* | 5’-TGCGACTTCAACAGCAACTC-3’ | 5’-CTTGCTCAGTGTCCTTGCTG-3’ |
| 12S rRNA | 5′‐TCTAGACATCCGTTTATGAGAGGAG‐3′ | 5′‐GGGTGTAGGCCAGATGCTTTAATA‐3′ |
| Actβ | 5′‐TTGCACATGCCGGAGCC‐3′ | 5′‐CGGCCCCGAGGTGACTAT‐3′ |
