# Supplementary material for: ZLN005 improves the survival of polymicrobial sepsis by increasing the bacterial killing via inducing lysosomal acidification and biogenesis in phagocytes
Source: Front Immunol. 2023 Feb 3;14:1089905. doi: 10.3389/fimmu.2023.1089905 (PMC9938763; doi:10.3389/fimmu.2023.1089905)
Supplement: Supplementary file 17 [file Table_2.docx]

**Supplement Table 2 |** Plasmid vector sequences.

| Plasmid vector | Sequence |
| --- | --- |
| pMXs-MTS-mKeima-Red | CCCGAAAAGTGCCACCTGCATAATGAAAGACCCCACCTGTAGGTTTGGCAAGCTAGCTTAAGTAACGCCATTTTGCAAGGCATGGAAAAATACATAACTGAGAATAGAAAAGTTCAGATCAAGGTCAGGAACAGATGGAACAGCTGAATATGGGCCAAACAGGATATCTGTGGTAAGCAGTTCCTGCCCCGGCTCAGGGCCAAGAACAGATGGAACAGCTGAATATGGGCCAAACAGGATATCTGTGGTAAGCAGTTCCTGCCCCGGCTCAGGGCCAAGAACAGATGGTCCCCAGATGCGGTCCAGCCCTCAGCAGTTTCTAGAGAACCATCAGATGTTTCCAGGGTGCCCCAAGGACCTGAAATGACCCTGTGCCTTATTTGAACTAACCAATCAGTTCGCTTCTCGCTTCTGTTCGCGCGCTTCTGCTCCCCGAGCTCAATAAAAGAGCCCACAACCCCTCACTCGGCGCGCCAGTCCTCCGATTGACTGAGTCGCCCGGGTACCCGTGTATCCAATAAACCCTCTTGCAGTTGCATCCGACTTGTGGTCTCGCTGTTCCTTGGGAGGGTCTCCTCTGAGTGATTGACTACCCGTCAGCGGGGGTCTTTCATTTGGGGGCTCGTCCGGGATCGGGAGACCCCTGCCCAGGGACCACCGACCCACCACCGGGAGGTAAGCTGGCCAGCAACTTATCTGTGTCTGTCCGATTGTCTAGTGTCTATGACTGATTTTATGCGCCTGCGTCGGTACTAGTTAGCTAACTAGCTCTGTATCTGGCGGACCCGTGGTGGAACTGACGAGTTCGGAACACCCGGCCGCAACCCTGGGAGACGTCCCAGGGACTTCGGGGGCCGTTTTTGTGGCCCGACCTGAGTCCAAAAATCCCGATCGTTTTGGACTCTTTGGTGCACCCCCCTAATAGGAGGGATATGTGGTTCTGGTAGGAGACGAGAACCTAAAACAGTTCCCGCCTCCGTCTGAATTTTTGCTTTCGGTTTGGGACCGAAGCCGCGCCGCGCGTCTTGTCTGCTGCAGCATCGTTCTGTGTTGTCTCTGTCTGACTGTGTTTCTGTATTTGTCTGAAAATTAGGGCCAGACTGTTACCACTCCCTTAAGTTTGACCTTAGGTCACTGGAAAGATGTCGAGCGGATCGCTCACAACCAGTCGGTAGATGTCAAGAAGAGACGTTGGGTTACCTTCTGCTCTGCAGAATGGCCAACCTTTAACGTCGGATGGCCGCGAGACGGCACCTTTAACCGAGACCTCATCACCCAGGTTAAGATCAAGGTCTTTTCACCTGGCCCGCATGGACACCCAGACCAGGTCCCCTACATCGTGACCTGGGAAGCCTTGGCTTTTGACCCCCCTCCCTGGGTCAAGCCCTTTGTACACCCTAAGCCTCCGCCTCCTCTTCCTCCATCCGCCCCGTCTCTCCCCCTTGAACCTCCTCGTTCGACCCCGCCTCGATCCTCCCTTTATCCAGCCCTCACTCCTTCTCTAGGCGCCCCCATATGGCCATATGAGATCTTATATGGGGCACCCCCGCCCCTTGTAAACTTCCCTGACCCTGACATGACAAGAGTTACTAACAGCCCCTCTCTCCAAGCTCACTTACAGGCTCTCTACTTAGTCCAGCACGAAGTCTGGAGACCTCTGGCGGCAGCCTACCAAGAACAACTGGACCGACCGGTGGTACCTCACCCTTACCGAGTCGGCGACACAGTGTGGGTCCGCCGACACCAGACTAAGAACCTAGAACCTCGCTGGAAAGGACCTTACACAGTCCTGCTGACCACCCCCACCGCCCTCAAAGTAGACGGCATCGCAGCTTGGATACACGCCGCCCACGTGAAGGCTGCCGACCCCGGGGGTGGACCATCCTCTAGACTGCCGGATCTAGCTAGTTAATTAAGGATCTGCCACCATGTCCGTCCTGACGCCGCTGCTGCTGCGGGGCTTGACAGGCTCGGCCCGGCGGCTCCCAGTGCCGCGCGCCAAGATCCATTCGTTGACGCGTATGGTGAGTGTGATCGCTAAACAAATGACCTACAAGGTTTATATGTCAGGCACGGTCAATGGACACTACTTTGAGGTCGAAGGCGATGGAAAAGGAAAGCCTTACGAGGGAGAGCAGACAGTAAAGCTCACTGTCACCAAGGGTGGACCTCTGCCATTTGCTTGGGATATTTTATCACCACAGCTTCAGTACGGAAGCATACCATTCACCAAGTACCCTGAAGACATCCCTGATTATTTCAAGCAGTCATTCCCTGAGGGATATACATGGGAGAGGAGCATGAACTTTGAAGATGGTGCAGTGTGTACTGTCAGCAATGATTCCAGCATCCAAGGCAACTGTTTCATCTACAATGTCAAAATCTCTGGTGAGAACTTTCCTCCCAATGGACCTGTTATGCAGAAGAAGACACAGGGCTGGGAACCCAGCACTGAGCGTCTCTTTGCACGAGATGGAATGCTGATAGGAAACGATTATATGGCTCTGAAGTTGGAAGGAGGTGGTCACTATTTGTGTGAATTTAAATCTACTTACAAGGCAAAGAAGCCTGTGAGGATGCCAGGGCGCCACGAGATTGACCGCAAACTGGATGTAACCAGTCACAACAGGGATTACACATCTGTTGAGCAGTGTGAAATAGCCATTGCACGCCACTCTTTGCTCGGTTGAGCGGCCGCCAGCACAGTGGTCGACGATAAAATAAAAGATTTTATTTAGTCTCCAGAAAAAGGGGGGAATGAAAGACCCCACCTGTAGGTTTGGCAAGCTAGCTTAAGTAACGCCATTTTGGAAGGCATGGAAAAATACATAACTGAGAATAGAGAAGTTCAGATCAAGGTCAGGAACAGATGGAACAGCTGAATATGGGCCAAACAGGATATCTGTGGTAAGCAGTTCCTGCCCCGGCTCAGGGCCAAGAACAGATGGAACAGCTGAATATGGGCCAAACAGGATATCTGTGGTAAGCAGTTCCTGCCCCGGCTCAGGGCCAAGAACAGATGGTCCCCAGATGCGGTCCAGCCCTCAGCAGTTTCTAGAGAACCATCAGATGTTTCCAGGGTGCCCCAAGGACCTGAAATGACCCTGTGCCTTATTTGAACTAACCAATCAGTTCGCTTCTCGCTTCTGTTCGCGCGCTTCTGCTCCCCGAGCTCAATAAAAGAGCCCACAACCCCTCACTCGGGGCGCCAGTCCTCCGATTGACTGAGTCGCCCGGGTACCCGTGTATCCAATAAACCCTCTTGCAGTTGCATCCGACTTGTGGTCTCGCTGTTCCTTGGGAGGGTCTCCTCTGAGTGATTGACTACCCGTCAGCGGGGGTCTTTCACATGCAGCATGTATCAAAATTAATTTGGTTTTTTTTCTTAAGTATTTACATTAAATGGCCATAGTTGCATTAATGAATCGGCCAACGCGCGGGGAGAGGCGGTTTGCGTATTGGGCGCTCTTCCGCTTCCTCGCTCACTGACTCGCTGCGCTCGGTCGTTCGGCTGCGGCGAGCGGTATCAGCTCACTCAAAGGCGGTAATACGGTTATCCACAGAATCAGGGGATAACGCAGGAAAGAACATGTGAGCAAAAGGCCAGCAAAAGGCCAGGAACCGTAAAAAGGCCGCGTTGCTGGCGTTTTTCCATAGGCTCCGCCCCCCTGACGAGCATCACAAAAATCGACGCTCAAGTCAGAGGTGGCGAAACCCGACAGGACTATAAAGATACCAGGCGTTTCCCCCTGGAAGCTCCCTCGTGCGCTCTCCTGTTCCGACCCTGCCGCTTACCGGATACCTGTCCGCCTTTCTCCCTTCGGGAAGCGTGGCGCTTTCTCATAGCTCACGCTGTAGGTATCTCAGTTCGGTGTAGGTCGTTCGCTCCAAGCTGGGCTGTGTGCACGAACCCCCCGTTCAGCCCGACCGCTGCGCCTTATCCGGTAACTATCGTCTTGAGTCCAACCCGGTAAGACACGACTTATCGCCACTGGCAGCAGCCACTGGTAACAGGATTAGCAGAGCGAGGTATGTAGGCGGTGCTACAGAGTTCTTGAAGTGGTGGCCTAACTACGGCTACACTAGAAGGACAGTATTTGGTATCTGCGCTCTGCTGAAGCCAGTTACCTTCGGAAAAAGAGTTGGTAGCTCTTGATCCGGCAAACAAACCACCGCTGGTAGCGGTGGTTTTTTTGTTTGCAAGCAGCAGATTACGCGCAGAAAAAAAGGATCTCAAGAAGATCCTTTGATCTTTTCTACGGGGTCTGACGCTCAGTGGAACGAAAACTCACGTTAAGGGATTTTGGTCATGAGATTATCAAAAAGGATCTTCACCTAGATCCTTTTGCGGCCGGCCGCAAATCAATCTAAAGTATATATGAGTAAACTTGGTCTGACAGTTACCAATGCTTAATCAGTGAGGCACCTATCTCAGCGATCTGTCTATTTCGTTCATCCATAGTTGCCTGACTCCCCGTCGTGTAGATAACTACGATACGGGAGGGCTTACCATCTGGCCCCAGTGCTGCAATGATACCGCGAGACCCACGCTCACCGGCTCCAGATTTATCAGCAATAAACCAGCCAGCCGGAAGGGCCGAGCGCAGAAGTGGTCCTGCAACTTTATCCGCCTCCATCCAGTCTATTAATTGTTGCCGGGAAGCTAGAGTAAGTAGTTCGCCAGTTAATAGTTTGCGCAACGTTGTTGCCATTGCTACAGGCATCGTGGTGTCACGCTCGTCGTTTGGTATGGCTTCATTCAGCTCCGGTTCCCAACGATCAAGGCGAGTTACATGATCCCCCATGTTGTGCAAAAAAGCGGTTAGCTCCTTCGGTCCTCCGATCGTTGTCAGAAGTAAGTTGGCCGCAGTGTTATCACTCATGGTTATGGCAGCACTGCATAATTCTCTTACTGTCATGCCATCCGTAAGATGCTTTTCTGTGACTGGTGAGTACTCAACCAAGTCATTCTGAGAATAGTGTATGCGGCGACCGAGTTGCTCTTGCCCGGCGTCAATACGGGATAATACCGCGCCACATAGCAGAACTTTAAAAGTGCTCATCATTGGAAAACGTTCTTCGGGGCGAAAACTCTCAAGGATCTTACCGCTGTTGAGATCCAGTTCGATGTAACCCACTCGTGCACCCAACTGATCTTCAGCATCTTTTACTTTCACCAGCGTTTCTGGGTGAGCAAAAACAGGAAGGCAAAATGCCGCAAAAAAGGGAATAAGGGCGACACGGAAATGTTGAATACTCATACTCTTCCTTTTTCAATATTATTGAAGCATTTATCAGGGTTATTGTCTCATGAGCGGATACATATTTGAATGTATTTAGAAAAATAAACAAATAGGGGTTCCGCGCACATTTC |
